# Termination codon readthrough of *NNAT* mRNA regulates calcium-mediated neuronal differentiation

**DOI:** 10.1016/j.jbc.2023.105184

**Published:** 2023-08-22

**Authors:** Madhuparna Pandit, Md Noor Akhtar, Susinder Sundaram, Sarthak Sahoo, Lekha E. Manjunath, Sandeep M. Eswarappa

**Affiliations:** 1 Department of Biochemistry, Indian Institute of Science, Bengaluru, Karnataka, India; 2 Undergraduate Program, Indian Institute of Science, Bengaluru, India

**Keywords:** stop codon, translational readthrough, neuronal differentiation, NNAT, SERCA2

## Abstract

Termination codon readthrough (TCR) is a process in which ribosomes continue to translate an mRNA beyond a stop codon generating a C-terminally extended protein isoform. Here, we demonstrate TCR in mammalian *NNAT* mRNA, which encodes NNAT, a proteolipid important for neuronal differentiation. This is a programmed event driven by *cis*-acting RNA sequences present immediately upstream and downstream of the canonical stop codon and is negatively regulated by NONO, an RNA-binding protein known to promote neuronal differentiation. Unlike the canonical isoform NNAT, we determined that the TCR product (NNATx) does not show detectable interaction with the sarco/endoplasmic reticulum Ca^2+^-ATPase isoform 2 Ca^2+^ pump, cannot increase cytoplasmic Ca^2+^ levels, and therefore does not enhance neuronal differentiation in Neuro-2a cells. Additionally, an antisense oligonucleotide that targets a region downstream of the canonical stop codon reduced TCR of *NNAT* and enhanced the differentiation of Neuro-2a cells to cholinergic neurons. Furthermore, NNATx-deficient Neuro-2a cells, generated using CRISPR-Cas9, showed increased cytoplasmic Ca^2+^ levels and enhanced neuronal differentiation. Overall, these results demonstrate regulation of neuronal differentiation by TCR of *NNAT*. Importantly, this process can be modulated using a synthetic antisense oligonucleotide.

*NNAT* is a maternally imprinted gene located on the opposite strand of the intron of the gene *BLCAP* (*q* arm of the chromosome 20 in humans). *NNAT* is expressed in neural, endocrine, and adipose tissues (1). The expression is particularly high in mammalian foetal and neonatal brain (2). Alternative splicing of *NNAT* pre-mRNA results in two proteolipid isoforms—neuronatin α (NNATα, 81 amino acids) and neuronatin β (NNATβ, 54 amino acids) (3).

NNAT is required for the efficient induction of neural lineage in embryonic stem cells. This induction is mediated by an increase in cytoplasmic Ca^2+^ level due to the inhibition of sarco/endoplasmic reticulum Ca^2+^-ATPase isoform 2 (SERCA2/ATP2A2) by NNAT (4). SERCA2, a P-type ATPase localized on the membrane of the endoplasmic reticulum, pumps Ca^2+^ from the cytoplasm to the lumen of endoplasmic reticulum, which serves as a store for these cations (5). Increase in cytoplasmic Ca^2+^ level upon NNAT overexpression has been observed in primary hippocampal neurons, HT22 hippocampal cell line, pancreatic cells, and 3T3-L1 preadipocytes (6, 7, 8). In pancreatic β-cells, NNAT overexpression increases insulin secretion (9). Mice and β-cells (NIT cell line) deficient in NNAT show reduced glucose-induced insulin secretion (10, 11). The expression of NNAT is increased in aortic endothelial cells of diabetic mice, which can activate proinflammatory signaling pathways. This function may have a role in the development of vascular complications found in diabetes (12). Knockdown of *NNAT* in primary subcutaneous white adipocytes results in browning, which aids in the disposal of excess stored lipid and reduction in insulin resistance (13). Thus, NNAT plays a crucial role in glucose metabolism, in addition to neuronal development.

Aberrant expression of, and mutations in *NNAT* are associated with several pathological conditions. Polymorphisms in *NNAT* are associated with childhood and adult obesity (14). Lafora disease, a progressive myoclonus epilepsy condition, is characterized by increased expression of *NNAT* in the brain cortex (15). High NNAT expression is linked to poor prognosis in breast cancer patients and glioblastoma patients (16, 17). Increased expression of NNAT is also implicated in the pathogenesis of Angelman syndrome, a neurodevelopmental disorder characterized by intellectual and motor disabilities (8). Thus, proper regulation of *NNAT* expression is important for the normal human physiology.

Regulation of *NNAT* expression has been observed at transcriptional and posttranscriptional levels. The transcription factor NeuroD1 (BETA2) binds the promoter region of *NNAT* and drives its expression in the pancreas (11). miRNA-708 inhibits the expression of *NNAT* by targeting its 3′ UTR. This pathway is suppressed in metastatic cancers (18). In the hypothalamus, *NNAT* expression is induced by leptin, a hormone that inhibits appetite (14).

Gene expression can also be regulated at translational level by recoding events, where the linear genetic information in the mRNA is read in alternative ways (19). For example, ribosomal frameshifting, where ribosomes change the translational frame (20). Termination codon readthrough (TCR) is another type of recoding process, where translating ribosomes recognize stop codons as sense codons. This results in continuation of translation beyond the stop codon generating a protein isoform with C-terminal extension. Normally, basal TCR is very low (21). However, *cis*- and *trans*-factors increase TCR to physiologically relevant frequency (>1%). More than ten mammalian mRNAs have been demonstrated to undergo TCR (22). This process can change the expression, localization, and/or function of the protein. Several cellular processes including angiogenesis, mitochondrial respiration, apoptosis, and myelination are regulated by TCR (23, 24, 25, 26, 27).

In this study, we show that the *NNAT* mRNA undergoes TCR, which generates a longer isoform termed NNATx with extra 89 amino acids (in humans) at the C-terminus. This process is negatively regulated by NONO, a nucleic acid binding protein. Unlike NNAT, NNATx does not interact with SERCA2, and therefore cannot increase cytoplasmic Ca^2+^ level. Because of this property, NNATx fails to induce neuronal differentiation, unlike NNAT. We also show that TCR of *NNAT* and neuronal differentiation can be modulated by an antisense oligonucleotide that targets the proximal 3′ UTR of *NNAT* mRNA.

## Results

### *NNAT* mRNA exhibits TCR

**Figure 1:**
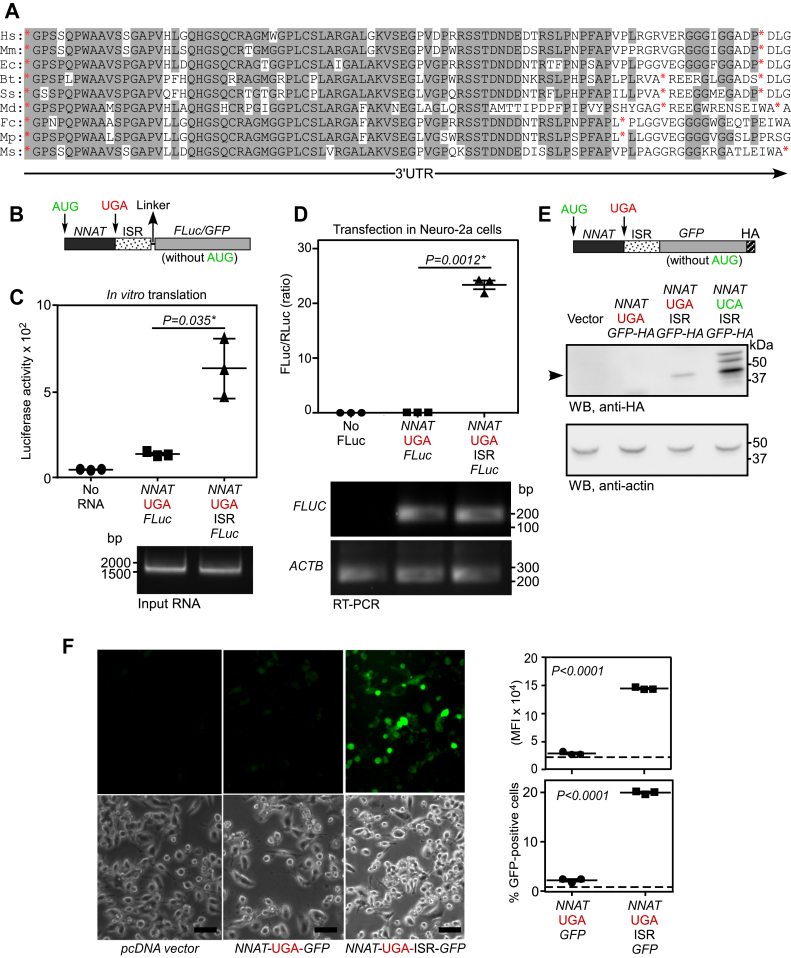
***NNAT* mRNA exhibits termination codon readthrough.***A*, alignment of amino acid sequences predicted from the proximal 3′ UTR of *NNAT* mRNA belonging to multiple mammalian species. Positions of stop codons are indicated in *red asterisks*. Conserved residues are shown in *gray background*. *B*, schematic of the construct used in the luminescence- and fluorescence-based assays. *C* and *D*, results of luminescence-based TCR assay performed *in vitro* using rabbit reticulocyte lysate (*C*) and by transfection in Neuro-2a cells (*D*). Input RNA and firefly luciferase mRNA levels by RT-PCR are shown below the graphs. *E*, detection of TCR product by Western blot. Cells were transfected with indicated plasmids (see schematic above) and Western blotting was performed 48 h after that. *Arrowhead* indicates the TCR product. The result is a representative of two independent experiments. *F*, results of fluorescence-based TCR assay performed in Neuro-2a cells. Images of Neuro-2a cells transfected with indicated plasmids taken in a fluorescence microscope are shown. The scale bar represents 50 μm. Quantification of mean fluorescence intensity (MFI) and % of GFP-positive cells using flow cytometry are also shown. *Dotted lines* indicate the values for vector-transfected cells. All graphs (mean ± SD) are representatives of at least three independent experiments performed with triplicate samples. Statistical significance was calculated using two-tailed Student’s *t* test (∗, Welch’s correction was applied). Hs, *Homo sapiens*; Mm, *Macaca mulatta*; Ec, *Equus caballus*; Bt, *Bos taurus*; Ss, *Sus scrofa*; Md, *Myotis davidii*; Fc, *Felis catus*; Mp, *Mustela putorius furo*. Ms, *Mus musculus*; TCR, termination codon readthrough.

Evolutionary conservation at the 3′ UTR of an mRNA is an indication of TCR (23, 28). This strategy was used to predict and demonstrate TCR in mammalian *AGO1* and *MTCH2* previously (23). *NNAT* is one such gene that showed evolutionary conservation in the 3′ UTR at nucleotide and predicted amino acid levels across mammals. Also, there is an in-frame stop codon downstream (267 and 276 nts in humans and mice, respectively) of the canonical stop codon (Figs. 1*A* and S1). Based on these observations, we hypothesized that *NNAT* mRNA exhibits TCR to generate a C-terminally extended isoform.

To test this hypothesis, we employed luminescence-based TCR assay as described before (25). We cloned the coding sequence (CDS) and the inter stop codon region (ISR, the region between the canonical stop codon and the downstream in-frame stop codon in the 3′UTR, Fig. S1) of *NNAT*, upstream of and in-frame with the CDS of firefly luciferase (FLuc) without its start codon (*NNAT*(*UGA*)-ISR-*FLUC*, Fig. 1*B*). Luciferase activity (*i.e.*, luminescence) was expected only if there was TCR across the canonical stop codon of the *NNAT*. The construct was subjected to *in vitro* transcription, followed by *in vitro* translation using rabbit reticulocyte lysate. We observed significant FLuc activity, much above the background level, from the *NNAT*(*UGA*)-ISR-*FLUC* construct. The activity was reduced to background level when the ISR region was removed from the construct (Fig. 1*C*). The constructs were transfected in Neuro-2a cells (neuroblast cell line) along with a plasmid expressing *Renilla* luciferase (RLuc) as a transfection control. The ratio of FLuc activity to the RLuc activity was quantified. We observed significant FLuc activity, much above the background level (0.01–0.1% in mammalian cells (21)) from the *NNAT*(*UGA*)-ISR-*FLUC* construct. The activity was reduced to background level when the ISR region was removed from the construct. However, the *FLUC* mRNA levels were comparable in both the constructs. These results indicate TCR across the canonical stop codon of *NNAT* mediated by its ISR (Fig. 1*D*). Furthermore, the readthrough product of the expected size (∼47 kDa with tags) was detectable by Western blotting in lysates of cells transfected with *NNAT*(*UGA*)-ISR-GFP-hemagglutinin reporter construct (Fig. 1*E*). This observation rules out translation reinitiation in the ISR.

Next, we employed fluorescence-based assay to test TCR in *NNAT*. The CDS of FLuc in the construct described above was replaced with that of the GFP such that fluorescence could be observed only if there was TCR across the canonical stop codon of *NNAT* (Fig. 1*B*). When transfected in Neuro-2a cells, we observed fluorescence in cells transfected with *NNAT*(*UGA*)-ISR-*GFP*. The mean fluorescence intensity and the percentage of fluorescent cells were reduced to background level, when the ISR was removed from the construct (Fig. 1*F*). Together, these results support our hypothesis that *NNAT* mRNA exhibits TCR.

**Figure 2:**
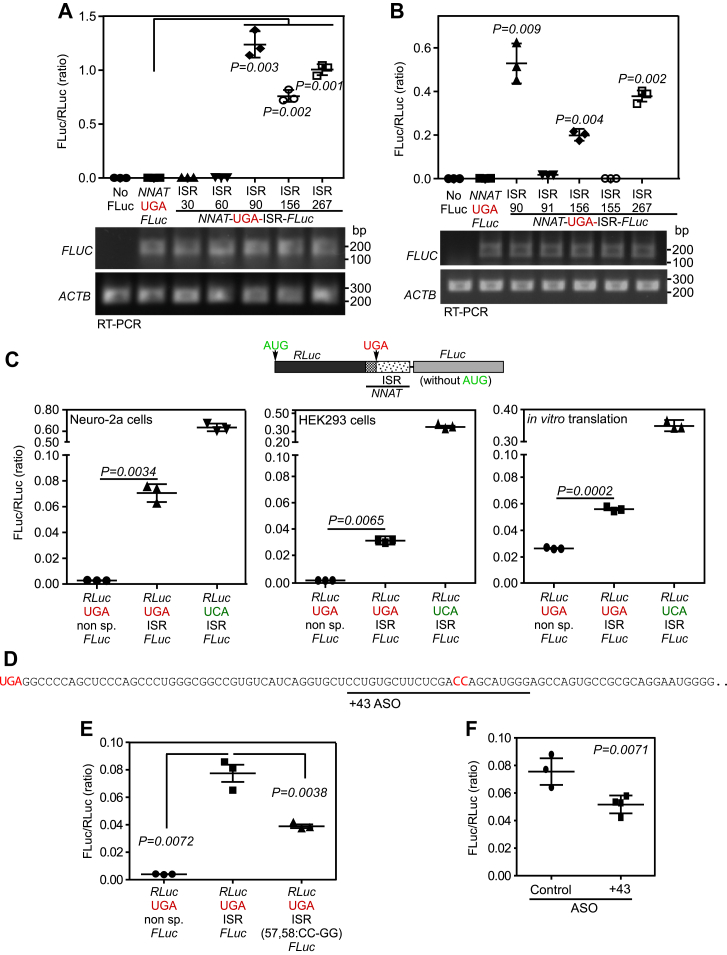
**Determinants of TCR of *NNAT* mRNA.** Results of luminescence-based TCR assay were performed in Neuro-2a cells, (*A*) to determine the stretch of ISR essential for TCR and (*B*) in constructs, where *NNAT* and *FLUC* are out-of-frame (see Fig. 1*B* for the schematic). Firefly luciferase mRNA levels by RT-PCR are shown below the graphs. ISR numbers indicate the length of the ISR sequence (from 5′) used in the assay. *C*, results of dual luciferase–based TCR assay performed in Neuro-2a cells, HEK293 cells, and *in vitro* using rabbit reticulocyte lysate. The *y*-axis indicates Fluc activity/Rluc activity (ratio) for each indicated construct. *D*, sequence of the 90 nucleotides (from the 5′ end) of the *NNAT* ISR. The region between 43rd to 66th nucleotides targeted by the +43 ASO is *underlined*. The canonical stop codon (UGA) and the mutated nucleotides (57th and 58th) are shown in *red*. *E*, result of dual luciferase–based TCR assay in Neuro-2a cells using a construct with mutated ISR (CC→GG in 57th and 58th positions). *F*, result of dual luciferase–based SCR assay performed in Neuro-2a cells transfected with control or +43 ASO. All graphs (mean ± SD) are representatives of three independent experiments performed with at least triplicate samples. Statistical significance was calculated using two-tailed Student’s *t* test with Welch’s correction. ASO, antisense oligonucleotide; ISR, inter stop codon region; TCR, termination codon readthrough.

To identify the minimum length of the ISR required for TCR, we made constructs with ISR of different lengths—30, 60, 90, 156, or 267 (full length) nucleotides (from the 5′ end)—in *NNAT*-*FLUC* background maintaining the same translation frame. Luminescence assay in Neuro-2a cells using these constructs revealed that the first 90 nucleotides of the ISR are important for TCR (Fig. 2*A*). We also generated *NNAT*-*FLUC* constructs such that *NNAT* and *FLUC* are out-of-frame by cloning 91 or 155 nucleotides (from 5′ end) of the ISR between them. Only background luminescence was observed when they were transfected in Neuro-2a cells (Fig. 2*B*). This result also strengthened our TCR hypothesis.

To further confirm TCR in *NNAT*, we employed dual luciferase-based luminescence assay. We cloned a part of the CDS (90 nucleotides immediately upstream of canonical stop codon) and the ISR of *NNAT* between the CDSs of *RLUC* and *FLUC* such that they are in same translation frame. While RLuc is expressed constitutively, FLuc could be expressed only if there was TCR across the canonical stop codon of *NNAT* (schematic in Fig. 2*C*). A construct with a nonspecific sequence between *RLUC* and *FLUC* was used to estimate the background signal for the assay. TCR assay was performed using these constructs in Neuro-2a cells, HEK293 cells, and *in vitro* using rabbit reticulocyte lysate. FLuc/RLuc ratio will indicate the TCR activity. We observed significant FLuc activity in the construct with the ISR, much above the background activity, demonstrating TCR driven by the ISR of *NNAT* (Fig. 2*C*).

Another construct without any stop codon between *RLUC* and *FLUC* was used to estimate the efficiency of TCR (29, 30). This construct expresses both FLuc and RLuc constitutively. FLuc/RLuc ratio resulting from this construct was used to normalize the FLuc/RLuc ratio, resulting from the test constructs and calculate the % of TCR. We observed 11.1%, 7.2%, and 16.5% TCR in Neuro-2a cells, HEK293 cells, and by *in vitro* translation, respectively. A construct without ISR showed only background activity. The TCR efficiency was reduced by ≈50%, when the 90 nucleotides from the CDS was not included and only the ISR was present (Fig. S2*A*).

These results show that ISR is sufficient to induce TCR. As several TCR events are induced by *cis*-acting RNA secondary structures, we investigated the possible structures in *NNAT* ISR using RNAalifold webserver. This online tool predicts possible minimum energy consensus structures in a given RNA sequence alignment (31). It predicted a stem–loop structure in the first 90 nucleotides of the ISR (Fig. S2*B*). However, mutations of the nucleotides at two sides (positions 43,44:CC→GG and 66,67:GA→CC) of this predicted stem structure did not alter the TCR activity suggesting that the predicted structure, even if it exists, does not play any role in driving the TCR (Fig. S2, *C* and *D*). However, mutations at the nucleotide positions 57 and 58 (CC→GG), which are not part of the predicted stem structure, reduced the TCR activity by about 50% (Fig. 2, *D* and *E*). To further investigate this, we used antisense oligonucleotide (ASO) approach, which has been shown to modulate translational recoding events including TCR (32, 33, 34, 35). An ASO that targets a region between 43 and 66 nucleotides (termed +43 ASO) in the ISR decreased the TCR (Fig. 2*F*). Together, these observations suggest that the sequence between 43rd and 66th nucleotides of the ISR are important for the TCR of *NNAT.*

### Evidence for TCR in endogenous *NNAT* mRNA

**Figure 3:**
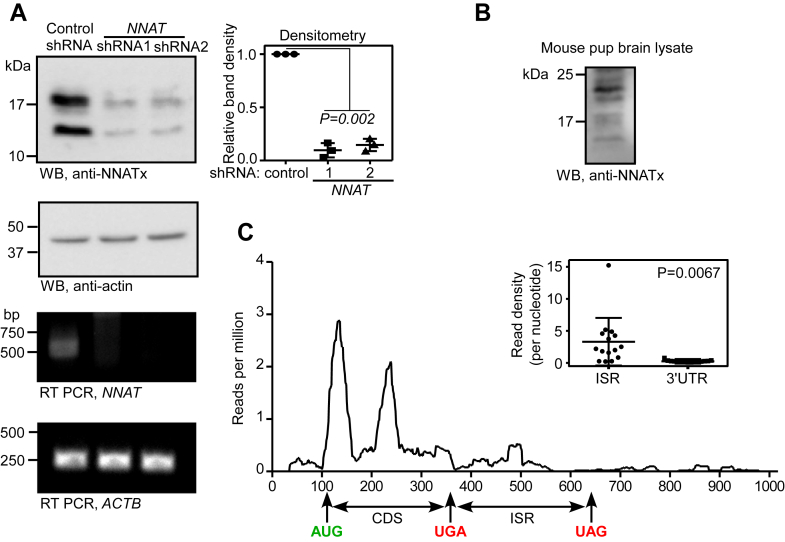
**TCR of endogenous *NNAT* mRNA.***A*, Western blot of lysates from Neuro-2a cells transfected with control shRNA or *NNAT*-targeting shRNAs performed using anti-NNATx antibody. Densitometry graph shows relative density (mean ± SD, *p* value by paired two-tailed Student’s *t* test) from three experiments. RT-PCR result showing *NNAT* mRNA levels in these cells is shown below. *B*, Western blot of mouse pup brain lysate showing the expression of NNATx *in vivo*. *C*, *NNAT* ribosome profile in mouse embryonic neural stem cells (SRR1382093) showing the presence of ribosomes in the ISR. Position of the start codon (*green*) and the two in-frame stop codons (*red*) are shown. Inset shows density of reads in the ISR and the 3′ UTR of mouse *NNAT* (N = 15 datasets). Statistical significance was calculated using two-tailed Student’s *t* test with Welch’s correction. TCR, termination codon readthrough.

After demonstrating TCR by exogenous expression of *NNAT*, we aimed to detect the endogenous TCR product of *NNAT* (termed NNATx). For this, we raised an antibody (anti-NNATx antibody) against the peptide, SSGAPVHLGQHGSQC, encoded in the ISR. This peptide sequence was chosen because it is unique to the ISR of *NNAT*. No other mammalian mRNA encodes this peptide sequence. Dot blot assay showed that NNATx antibody specifically detects this peptide (Fig. S3*A*). We then performed Western blot analysis of Neuro-2a lysate using this antibody. The assay detected two bands—one near 17 kDa and another one between 10 and 17 kDa. These molecular weights are consistent with the expected sizes of the TCR products of the two alternatively spliced *NNAT* mRNAs, that is, 170 (81 + 89) and 143 (54 + 89) amino acids. Importantly, the intensity of the bands reduced significantly in cells transfected with *NNAT*-targeting shRNAs (Fig. 3*A*). The band intensity was also reduced when the anti-NNATx antibody was preincubated with the peptide SSGAPVHLGQHGSQC (Fig. S3*B*). Furthermore, we could visualize the endogenous NNATx using anti-NNATx antibody in Neuro-2a cells by immunofluorescence microscopy. The fluorescent signal reduced to background level when the antibody was preincubated with the peptide SSGAPVHLGQHGSQC (Fig. S3*C*). NNATx was detected in the brain tissue sample from mouse pups, suggesting TCR of *NNAT in vivo* (Fig. 3*B*). Furthermore, expression of NNATx was observed in nonneuronal organs such as heart, liver, lung, kidney, and muscle (Fig. S3*D*).

Ribosome profiling is a technique that identifies mRNA regions undergoing translation. This technique is based on deep sequencing of ribosome protected RNA fragments. This method has been successfully used to identify novel TCR candidate mRNAs (36, 37). We analyzed ribosome profiling datasets derived from mouse embryos available in National Center for Biotechnology Information’s Sequence Read Archive (38, 39). Ribosome footprints in the ISR of *NNAT* were observed in mouse neuronal stem cells, mouse neural tubes, forelimb buds, and in primary cells derived from the primitive streak. The number and the density of reads corresponding to the ISR were much above the background level (observed in the rest of the 3′ UTR) (Figs. 3*C* and S4). Together, these results provide evidence for TCR of *NNAT in vitro* as well as *in vivo*.

### Non-POU domain–containing octamer-binding protein negatively regulates TCR of *NNAT* mRNA

*Trans*-acting factors such as proteins, miRNAs, and even synthetic molecules regulate the process of translational recoding events by interacting with target mRNAs. miR-1224 interacts with *CCR5* mRNA triggering −1 ribosomal frameshifting (40). Let-7a miRNA promotes TCR of *AGO1* mRNA (25). hnRNPA2/B1 protein binds the ISR of *VEGFA* and promotes its TCR (23). Annexin A2 protein reduces the frameshifting efficiency in infectious bronchitis virus (41). The viral protein Tat modulates −1 ribosomal frameshifting in HIV-1 (42). To identify such a factor for *NNAT*, if any, we used RBPsuite, a deep learning-based webserver to predict RNA-binding proteins (RBPs) for a given RNA sequence (43). The sequence of the first 101 nucleotides of the ISR (ISR^1-101^), which is critical for TCR in *NNAT* was given as input to predict RBPs. To eliminate nonspecific RBPs, we used the 3′ 101 nucleotides of the CDS (CDS^101^) and 101 nucleotides from the second half of the *NNAT* ISR (ISR^102-202^) as inputs. We searched for RBPs that bind ISR^1-101^, but not CDS^101^ or ISR^102-202^. This analysis predicted NONO as a unique RBP that can potentially bind the ISR of *NNAT* (Fig. S5*A*).

**Figure 4:**
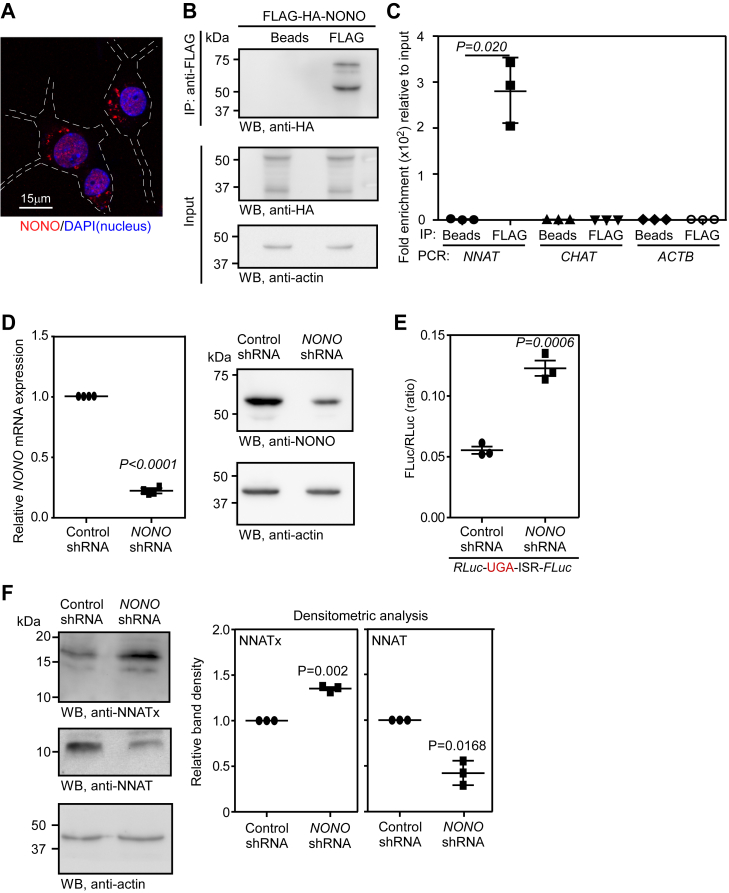
**NONO protein downregulates the TCR of *NNAT* mRNA.***A*, confocal fluorescence microscopy image of Neuro-2a cells showing the nuclear and cytoplasmic localization of NONO. Anti-NONO antibody was used to detect NONO. *White dashed lines* indicate the cell boundary inferred from the corresponding bright field image. *B*, Western blot showing the immunoprecipitation of FLAG-HA-NONO using anti-FLAG M2 affinity gel from transfected Neuro-2a cells. *C*, quantitative real-time PCR result showing the enrichment of *NNAT* mRNA in the samples shown in (*B*). *CHAT* and *ACTB* mRNAs were used as negative controls. *D*, quantitative real-time PCR result and Western blot showing reduced *NONO* expression in shRNA-transfected Neuro-2a cells. *E*, result of dual luciferase–based TCR assay showing increased *NNAT* readthrough in *NONO* shRNA-transfected Neuro-2a cells. The schematic of the construct used in this assay is shown in Figure 2*C*. *F*, Western blot showing the expression of NNATx and NNAT in shRNA-transfected Neuro-2a cells. Densitometric analyses of the band intensities are shown. Graphs (mean ± SD) and blots are representatives of three independent experiments. Statistical significance was calculated using unpaired (*C* and *E*) or paired (*D* and *F*) two-tailed Student’s *t* test. TCR, termination codon readthrough.

NONO (also known as p54nrb) belongs to *Drosophila* behaviour/human splicing protein family. It has two tandem RNA recognition motifs near the N-terminus. NONO regulates transcription, mRNA splicing, mRNA transport, and DNA repair (44). Though primarily a nuclear protein, it is found in neuronal cytoplasmic RNA granules (45, 46). Confocal microscopy revealed the cytoplasmic presence of NONO in Neuro-2a cells as well (Fig. 4*A*). To test the interaction of NONO with *NNAT* mRNA, we expressed FLAG-HA–tagged NONO in Neuro-2a cells and immunoprecipitated it using anti-FLAG M2 agarose beads (Fig. 4*B*). As predicted, the endogenous *NNAT* mRNA was enriched along with the immunoprecipitated NONO suggesting their interaction, which could be direct or indirect through other proteins. This was observed by both quantitative real-time PCR and semiquantitative PCR (Figs. 4*C* and S5*B*). Choline acetyltransferase (*CHAT*) and *ACTIN* mRNAs did not show this enrichment. To identify the potential interaction site, Neuro-2a cells were transfected with FLAG-HA–tagged NONO construct and NNAT constructs with multiple deletions in the ISR. NONO was immunoprecipitated using anti-FLAG beads, and the precipitate was used for RNA extraction, followed by RT-PCR of *NNAT-FLUC* mRNA. This analysis revealed that the region between 30 and 60 nucleotides in the ISR of *NNAT* is involved in the interaction with NONO (Fig. S5*C*). Notably, as shown above, the +43 ASO, which targets this region, reduced the TCR of *NNAT* (Fig. 2*F*). This suggested a similar regulatory role for NONO in the TCR of *NNAT*.

To understand the role of NONO in the TCR of *NNAT*, we generated stable Neuro-2a cells with shRNA-mediated *NONO* knockdown (Fig. 4*D*). In these cells we performed dual luciferase-based TCR assay for *NNAT* mRNA (schematic in Fig. 2*C*). We observed significant increase in the TCR in cells with *NONO* knockdown (Fig. 4*E*). In agreement with this, Western blot analysis revealed increased NNATx expression and reduced NNAT expression in these cells (Fig. 4*F*). These results together suggest that NONO is a negative regulator of TCR of *NNAT*.

### Unlike NNAT, NNATx does not increase cytoplasmic Ca^2+^ level

**Figure 5:**
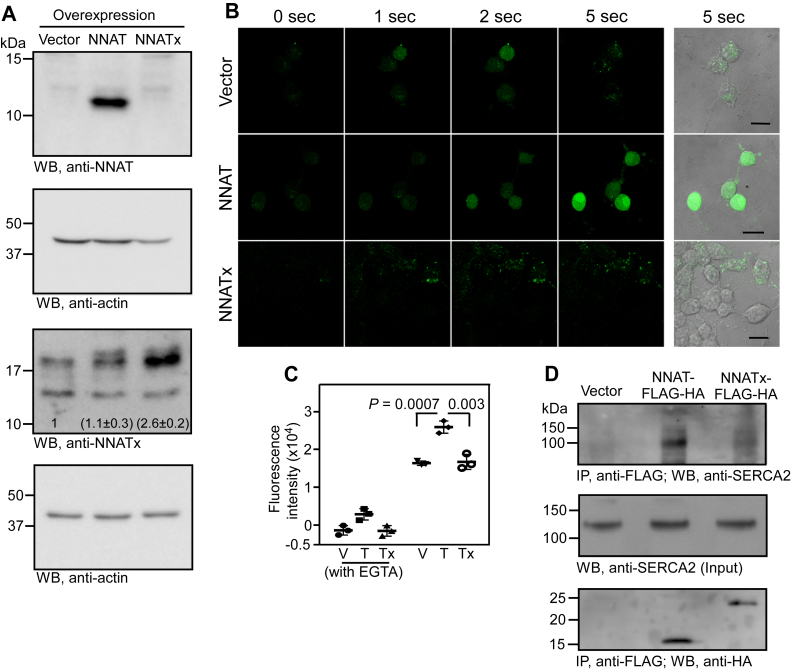
**Role of NNATx in the maintenance of cytoplasmic Ca**^**2+**^**level.***A*, Western blot showing the overexpression of NNAT and NNATx in Neuro-2a cells. Numbers in the NNATx blot indicate densitometry values (mean ± SD, n = 3, *p* = 0.004, paired two-tailed Student’s *t* test). *B*, confocal microscopy images showing cytoplasmic Ca^2+^ influx after adding 2 μM retinoic acid to Neuro-2a cells overexpressing NNAT or NNATx. The Scale bar represents 20 μm. Fluo-4 AM fluorescence (*green*) indicates cytoplasmic Ca^2+^ level. Images were acquired under identical microscope settings. This is a representative of two independent experiments. *C*, cytoplasmic Ca^2+^ level in NNAT- and NNATx-overexpressing Neuro-2a cells represented as fluorescence intensity. Cells were treated with differentiation medium containing 2 μM retinoic acid for 24 h and the cytoplasmic Ca^2+^ level was measured using Fluo-4 AM. The graph (mean ± SD) is a representative of three independent experiments performed with triplicate samples. Statistical significance was calculated using two-tailed Student’s *t* test. *D*, coimmunoprecipitation assay to test the interaction of NNAT-FLAG-HA and NNATx-FLAG-HA with SERCA2. A representative of two independent experiments is shown. Results of similar experiment performed in HEK293 cells are shown in Fig. S6*A*. SERCA2, sarco/endoplasmic reticulum Ca^2+^-ATPase isoform 2; T, NNAT; Tx, NNATx; V, Vector.

NNAT interacts with SERCA2 and inhibits its function resulting in increased cytoplasmic Ca^2+^ level (4). Overexpression of NNAT has been shown to increase cytoplasmic Ca^2+^ level in several cell types including neuronal cells (6, 7, 8). To understand the functional significance of TCR of *NNAT*, we investigated the effect of NNATx overexpression on the cytoplasmic Ca^2+^ level. We generated stable Neuro-2a cells that overexpress NNAT (81 amino acids) or NNATx (170 amino acids), which was confirmed by Western blotting (Fig. 5*A*). The anti-NNAT antibody did not recognize NNATx as expected from an antibody raised against the C-terminus.

Imaging of the cytoplasmic Ca^2+^ using Fluo-4 AM, a cell-permeant, green-fluorescent, high-affinity calcium indicator, immediately after retinoic acid addition showed enhanced Ca^2+^ influx within seconds in NNAT-overexpressing cells, but not in NNATx-overexpressing cells (Fig. 5*B*) (47, 48). We then quantified the cytoplasmic Ca^2+^ level. Cells treated with EGTA, a selective calcium ion chelator, were used to ensure that the fluorescent signal in our assay was due to Ca^2+^. We observed an increase in the Fluo-4 signal in NNAT-overexpressing cells. However, this increase was not observed in cells overexpressing NNATx (Fig. 5*C*).

To understand the mechanism behind this observation, we investigated the interaction of NNATx with SERCA2 in Neuro-2a cells. Consistent with previous reports, we could immunoprecipitate SERCA2 along with NNAT-FLAG-HA. However, SERCA2 was hardly detectable along with immunoprecipitated NNATx-FLAG-HA (Fig. 5*D*). This observation was made in HEK293 cells as well (Fig. S6*A*). These results show that NNATx does not exhibit detectable interaction with SERCA2, and therefore cannot increase the cytoplasmic Ca^2+^ level.

### Unlike NNAT, NNATx does not induce neuronal differentiation

**Figure 6:**
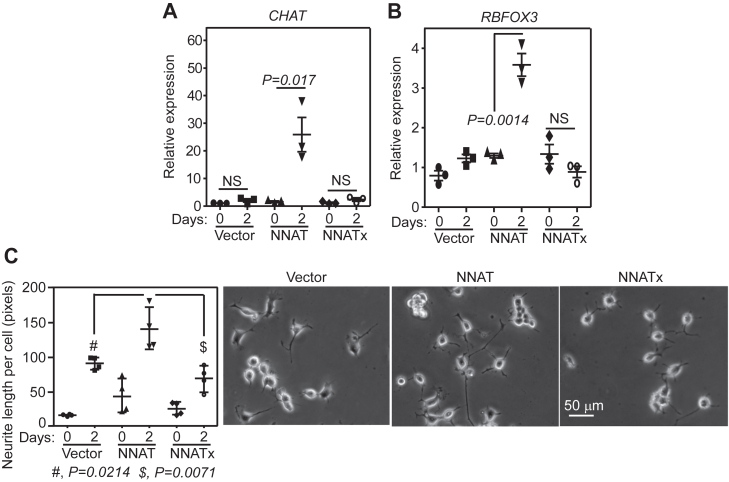
**NNATx does not induce neuronal differentiation.***A* and *B*, qRT-PCR results showing the expression of *CHAT* (*A*) and *RBFOX3* (*B*) mRNAs relative to *ACTB* mRNA in Neuro-2a cells overexpressing NNAT or NNATx. The graphs (mean ± SD) are representatives of three independent experiments performed with triplicate samples. Statistical significance was calculated using two-tailed Student’s *t* test. *C*, quantification of neurites length (mean ± SD) in Neuro-2a cells overexpressing NNAT or NNATx. At least 50 cells in each group from four biological replicates were analyzed to quantify the neurites. Representative images are shown. Cells were treated with differentiation medium containing 2 μM retinoic acid for 2 days. NS, not significant; CHAT, choline acetyltransferase.

Ca^2+^-mediated signaling is important for the differentiation of neural progenitor cells into neurons (49, 50). Hence, we investigated the role of NNATx in neuronal differentiation. Neuro-2a cells, like other neuroblast cells, can be differentiated into cholinergic neurons (51, 52). We tested the ability of NNAT- and NNATx-overexpressing Neuro-2a cells to differentiate into cholinergic neurons after retinoic acid treatment by quantifying the expression of *CHAT* mRNA, which is a marker for cholinergic neurons. We observed robust expression of *CHAT* mRNA in NNAT-overexpressing cells 48 h after retinoic acid treatment. However, NNATx-overexpressing cells did not exhibit any significant change in *CHAT* mRNA expression (Fig. 6*A*). Similarly, NNAT-overexpressing cells exhibited robust expression of *RBFOX3* (also known as *NEUN*), a neuronal marker, upon differentiation, which was not observed in NNATx-overexpressing cells (Fig. 6*B*) (53). We also measured neurite length, which is another indicator of differentiation. While NNAT-overexpressing cells showed much increased neurite length per cell, NNATx-overexpressing cells did not show this effect. Their average neurite length per cell was comparable to that of vector-transfected cells (Fig. 6*C*).

### NNATx-deficient cells exhibit increased differentiation

**Figure 7:**
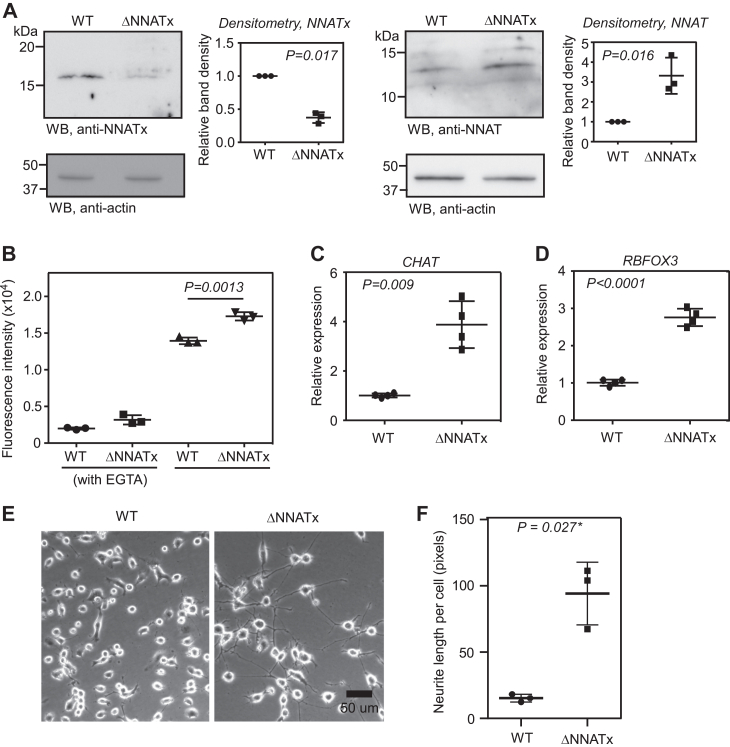
**NNATx deficient cells show enhanced differentiation.***A*, Western blot showing the levels of NNATx and NNAT in NNATx-deficient (ΔNNATx) Neuro-2a cells. The graphs show densitometric analysis (mean ± SD). *B*, cytoplasmic Ca^2+^ level in ΔNNATx Neuro-2a cells represented as fluorescence intensity. Cells were treated with differentiation medium for 24 h and the cytoplasmic Ca^2+^ level was measured using Fluo-4 AM. The graph (mean ± SD) is a representative of three independent experiments performed with triplicate samples. *C* and *D*, qRT-PCR results showing the expression of *CHAT* and *RBFOX3* mRNAs relative to *ACTB* mRNA in ΔNNATx Neuro-2a cells after 4 days of differentiation. Results are representatives of three independent experiments. *E*, images of WT and ΔNNATx Neuro-2a cells showing neurites after 4 days of differentiation. *F*, quantification of neurites length (mean ± SD, n = 3 fields). At least 65 cells in each group were analyzed to quantify the length of neurites. WT Neuro-2a cells. Statistical significance was calculated using two-sided paired (*A*, *C*, and *D*) or unpaired (*B* and *F*) two-tailed Student’s *t* test. ∗, Welch’s correction was applied. CHAT, choline acetyltransferase; qRT-PCR, quantitative real-time PCR.

To further confirm the significance of TCR of *NNAT* in neuronal differentiation, we generated NNATx-deficient Neuro-2a cells using CRISPR-Cas9 system (Fig. S7). These cells (ΔNNATx) showed reduced NNATx expression. The residual expression of NNATx could be due to more than two copies of chromosomes present in Neuro-2a cells. These cells also showed a significant increase in the expression of NNAT (Fig. 7*A*). This is expected as the *NNAT* mRNA can generate more canonical protein (NNAT). In agreement with this observation, ΔNNATx cells showed increased cytoplasmic Ca^2+^ levels (Fig. 7*B*), enhanced differentiation as indicated by increased expression of *CHAT* and *RBFOX3* genes (Fig. 7, *C* and *D*), and more neurite formation (Fig. 7, *E* and *F*). Together, these results provide more evidence to propose TCR of *NNAT* as a negative regulator of neuronal differentiation.

### TCR of *NNAT* and neuronal differentiation can be modulated using ASOs

**Figure 8:**
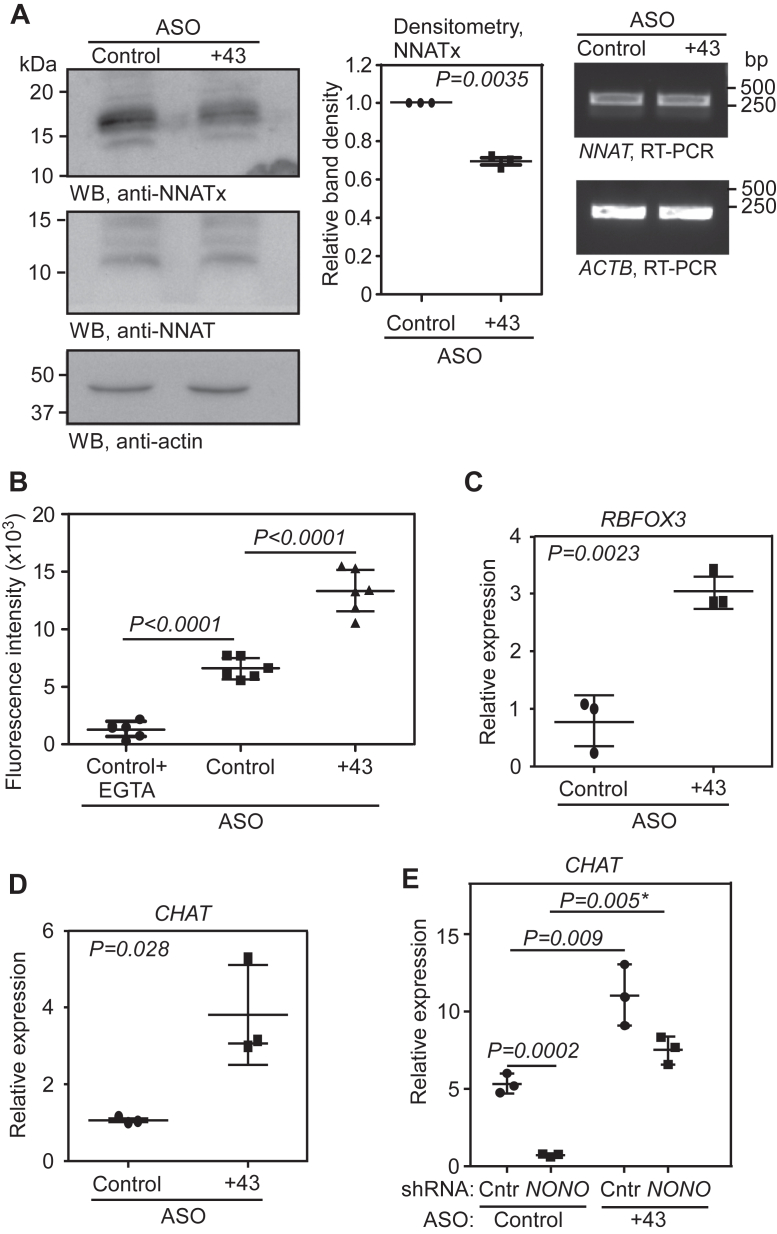
**Exogenous modulation of neuronal differentiation using *NNAT*-targeting antisense oligonucleotide**. *A*, Western blot showing the expression of NNATx and NNAT in cells transfected with control or +43 ASO. Quantification (mean ± SD) of the band intensities from three independent experiments is shown in the graphs. RT-PCR results show the levels of *NNAT* mRNA in ASO-transfected cells. *B*, cytoplasmic Ca^2+^ level in control- or +43 ASO-transfected Neuro-2a cells represented as fluorescence intensity. Cells were treated with differentiation medium containing 2 μM retinoic acid for 24 h and the cytoplasmic Ca^2+^ level was measured using Fluo-4 AM. The graph (mean ± SD) is a representative of two independent experiments performed with six samples in each set. *C* and *D*, qRT-PCR results showing the expression of *CHAT* and *RBFOX3* mRNAs relative to *ACTB* mRNA in Neuro-2a cells transfected with control or +43 ASO. Transfected cells were treated with differentiation medium containing 2 μM retinoic acid for 24 h before qRT-PCR was performed. *E*, qRT-PCR results showing the expression of *CHAT* relative to *ACTB* mRNA in Neuro-2a cells transfected with combinations of shRNAs and ASOs as indicated. Graphs (mean ± SD) represent results of three independent experiments performed with triplicate samples. Statistical significance was calculated using two-sided paired (*A*, *C*, and *D*) or unpaired (*B* and *E*) two-tailed Student’s *t* test. ∗, Welch’s correction was applied. CHAT, choline acetyltransferase; qRT-PCR, quantitative real-time PCR.

As shown above, +43 ASO was able to reduce the TCR of *NNAT* in the dual luciferase–based readthrough assay. We used this as a tool to modulate the level of endogenous NNATx. When transfected in Neuro-2a cells, +43 ASO was able to modestly reduce endogenous NNATx level compared to a control ASO that targets *GFP* (Fig. 8*A*). This effect was specific to +43 ASO, as ASOs that target other regions of the ISR (+1, +9, and +54; numbers indicate nucleotide position in the ISR, where the 5′ end of the ASO will bind) did not show any observable effect on NNATx levels (Fig. S8*A*). We confirmed the intracellular localization of fluorescently (Cy3) labeled +43 ASO after transfection in Neuro-2a cells by fluorescence microscopy and flow cytometry (Fig. S8, *B* and *C*). We then transfected Neuro-2a cells with control and +43 ASOs and measured the cytoplasmic Ca^2+^ level using Fluo-4 AM. There was a significant increase in the cytoplasmic Ca^2+^ level in cells treated with the +43 ASO compared to control ASO-treated cells (Fig. 8*B*). We also investigated the effect of the ASO on the differentiation of Neuro-2a cells. Cells treated with the +43 ASO showed much higher expression of *CHAT* and *RBFOX3* in differentiation medium after 24 h (Fig. 8, *C* and *D*).

Finally, +43 ASO was used to investigate the significance of NONO-mediated regulation of *NNAT* TCR. Neuro-2a cells showed reduced cholinergic differentiation when *NONO* was knocked down. Notably, +43 ASO could rescue this differentiation defect observed in *NONO* knockdown cells (Fig. 8*E*). These observations provide more evidence to propose that NONO-mediated regulation of *NNAT* TCR contributes to neuronal differentiation in Neuro-2a cells.

## Discussion

TCR is a process that generates more than one protein from the same mRNA. This serves as a mechanism to swiftly regulate the expression and/or function of a gene. In this study, we demonstrate TCR of *NNAT* mRNA using multiple methods: reporter-based assays, Western blotting and ribosome profiling data analyses (Figs. 1 and 3). The efficiency of TCR promoted by the *cis*-acting elements in the *NNAT* mRNA is approximately 11% based on the dual luciferase assay in Neuro-2a cells. This efficiency is much above the basal level of TCR, which is 0.01% to 0.1% (21). The first 90 nucleotides of the ISR are necessary for this process (Fig. 2). Thus, TCR of *NNAT* is a programmed translational event.

With respect to regulation of TCR, *NNAT* is similar to *VEGFA* and *AGO1*, whose TCR processes are regulated by *trans*-acting factors. The TCR of *VEGFA* is positively regulated by an RBP, hnRNPA2/B1 (23, 54); Let-7a miRNA upregulates the TCR of *AGO1* mRNA (25). hnRNPA2/B1 and Let-7a miRNA bind *VEGFA* and *AGO1* mRNAs, respectively, 10 nucleotides after the canonical stop codon. However, the possible NONO interaction site (direct or indirect) is present between 30th and 60th nucleotides on *NNAT* ISR (Fig. S5). This difference in the location of their binding sites on the corresponding mRNAs may be the reason why hnRNPA2/B1 and Let-7a miRNA are positive regulators of TCR, whereas NONO is a negative regulator. Previous reports have shown that NONO functions as a part of a multiprotein complex, which is important in RNA splicing, transport, and nuclear retention of abnormally edited RNAs (46, 55, 56). It is quite possible that NONO is part of a protein complex that interacts with *NNAT* mRNA (44). Further investigations are required to confirm these hypotheses and to understand the mechanism behind the negative effect of NONO on the TCR of *NNAT* mRNA.

**Figure 9:**
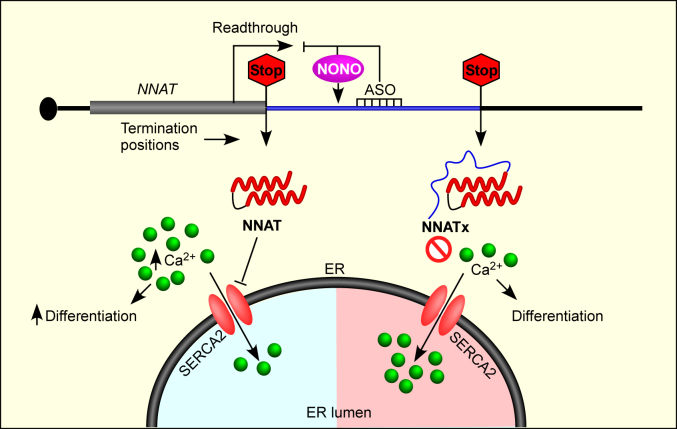
**The TCR of *NNAT* mRNA regulates cytoplasmic Ca**^**2+**^**level and neuronal differentiation.***NNAT* mRNA has two in-frame stop codons. Translation termination at the canonical stop codon generates NNAT isoform, which interacts with SERCA2 pump on the endoplasmic reticulum (ER) membrane and inhibits its function. This increases cytoplasmic Ca^2+^ level promoting neuronal differentiation. TCR at the canonical stop codon and termination at the downstream in-frame stop codon results in a C-terminally extended isoform termed NNATx. This process is negatively regulated by the protein NONO. NNATx has a predicted intrinsically disordered region at the C-terminus and it does not interact with SERCA2. Therefore, it cannot increase the cytoplasmic Ca^2+^ level and does not promote neuronal differentiation, unlike NNAT. TCR of *NNAT* can be inhibited, and neuronal differentiation can be enhanced, by a synthetic antisense oligonucleotide (ASO) that targets the ISR of *NNAT*. ISR, inter stop codon region; SERCA2, sarco/endoplasmic reticulum Ca^2+^-ATPase isoform 2; TCR, termination codon readthrough.

Unlike NNAT (the canonical isoform), NNATx (the product of TCR) does not exhibit detectable interaction with SERCA2, a Ca^2+^ pump on the endoplasmic reticulum. This could be due to the intrinsically disordered region encoded by the ISR after the TCR in *NNAT* mRNA (Fig. S6, *B*–*E*). Because of this property, NNATx overexpression does not result in an increase in cytoplasmic Ca^2+^ level and neuronal differentiation (Fig. 5). Notably, like *NNAT*, TCR of *VEGFA*, *VDR*, and *AGO1* result in isoforms with compromised canonical functions (23, 25, 57). Thus, TCR seems to be a mechanism to quickly tune down the function of a gene in mammals (Fig. 9).

Interestingly, NONO also plays a key positive role in the neuronal differentiation of embryonic stem cells (58). This is attributed to its role in the transcription of certain genes required during differentiation. Inhibition of *NNAT* TCR by NONO also contributes to this effect. This is supported by our observation that reduced differentiation of *NONO* knockdown cells was partially rescued by inhibiting *NNAT* TCR by a specific ASO (Fig. 8*E*). Our experiments also demonstrate that the TCR in *NNAT* can be inhibited using this ASO which targets the ISR from 43rd to 66th nucleotides. This effect leads to increased cytoplasmic Ca^2+^ level and enhanced differentiation of the mouse neuroblast cells into cholinergic neurons. This approach can be potentially used to treat neuronal disorders characterized by degeneration of cholinergic neurons such as Alzheimer’s disease. In fact, choline esterase inhibitors such as donepezil are used in the management of this condition (59). NNAT is also involved in the secretion of insulin from pancreatic β cells. The ASO-mediated inhibition of TCR in *NNAT* mRNA can be potentially used in the treatment of insulin-dependent diabetic conditions.

## Experimental procedures

### Cell culture

Neuro-2a cells (American Type Culture Collection) were cultured in Minimum Essential Medium Eagle (MEM, Sigma) supplemented with 1 mM sodium pyruvate (Sigma), 10% fetal bovine serum (FBS, Gibco), and 1% antibiotics (10,000 U/ml penicillin, 10,000 μg/ml streptomycin). Cells were maintained at 37 °C in a humidified atmosphere with 5% CO_2_. Cells were subjected to *mycoplasma* detection test at least once in 6 months to rule out *mycoplasma* contamination. For differentiation, Neuro-2a cells were maintained in MEM containing 1 mM sodium pyruvate, 0.5% FBS, 1% antibiotics, and 2 μM retinoic acid (Sigma). For differentiation experiments involving ΔNNATx cells, retinoic acid was not included in the differentiation medium as it showed toxicity in these mutant cells.

### Antibodies

The polyclonal anti-NNATx antibody was raised by injecting the synthetic peptide SSGAPVHLGQHGSQCR in rabbits. The same peptide was used for affinity purification (ABGENEX). Anti-NNAT (Abcam, ab27266), anti-HA (Sigma, 11867423001), anti-NONO (Santa Cruz Biotechnology, SC-166702), anti-SERCA2 (Cell Signaling Technologies, 4388S), anti-actin (Sigma, A3854), and secondary antibodies conjugated with horseradish peroxidase (Thermo Fisher Scientific) or Alexa Fluor (Molecular Probes) were used at dilutions recommended by the manufacturers.

### TCR assay

Cells were seeded at 70% to 90% confluency in 24-well plates. Plasmids (FLuc constructs, 500 ng/well and RLuc construct, 100 ng/well) were transfected into these cells using Lipofectamine 2000 (Invitrogen) following manufacturer’s protocol. Cells were lysed 24 h post transfection. FLuc and RLuc activities were measured using Dual-Luciferase Reporter Assay System (Promega) in GloMax Explorer System (Promega). For fluorescence-based readthrough assay, cells were analyzed by fluorescence microscopy (Olympus IX73) and flow cytometry (CytoFLeX S, Beckman Coulter) 24 h post transfection.

For *in vitro* translation, 2 μg of the luciferase reporter construct was linearized using NotI enzyme (Thermo Fisher Scientific). The linear plasmid was *in vitro* transcribed using T7 RNA polymerase (Invitrogen) at 37 °C for 2 h. Two micrograms of the *in vitro* transcribed RNA was then *in vitro* translated using rabbit reticulocyte system (Promega) at 30 °C for 2 h following manufacturer’s protocol. Luciferase activity was measured as described above.

% TCR was calculated using the following formula:

{[mean $\frac{Fluc}{RLuc}$ of “with-stop” construct]/[mean $\frac{Fluc}{RLuc}$ of “no-stop” construct]} ∗ 100

Neuro-2a cells: (0.0705/0.6347) ∗ 100 = 11.1%

HEK293 cells: (0.0273/0.3748) ∗ 100 = 7.2%

*In vitro* translation: (0.0576/0.3488) ∗ 100 = 16.5%

The mean values required for these calculations were taken from the data shown in Figure 2*C*.

### RNA isolation and RT-PCR

Total RNA from cells was isolated using TRI Reagent (Sigma) or RNAiso Plus (Takara) following the manufacturers’ protocol. Oligo-dT universal reverse primer was used for complementary DNA synthesis with RevertAid Reverse Transcriptase (Invitrogen) or M-MuLV Reverse Transcriptase (New England BioLabs). Semiquantitative PCR was done using gene-specific primers and Taq DNA polymerase (Takara).

For qRT-PCR analysis, complementary DNA synthesized from 2 μg total RNA was diluted five times. PCR amplification was carried out using gene-specific primers and TB Green Premix Ex Taq II (Takara), following the manufacturer’s protocol on CFX96 Real-Time PCR Detection System (Bio-Rad). Following are the cycling conditions: 95 °C for 5 min, followed by 40 cycles of 95 °C for 30 s, 56 °C (*CHAT*) or 54 °C (*RBFOX3*) for 30 s, 72 °C for 30 s, and a final extension step at 72 °C for 5 min. After these amplification steps, they were heated at 55 °C for 20 s, followed by gradual cooling from 95 °C to 0.5 °C to generate melt curves. The amplified PCR products were also resolved by agarose gel electrophoresis to confirm the absence of nonspecific amplifications. Gene expression was quantified relative to the expression of *ACTB* using delta–delta Ct (2^-ΔΔCt^) method.

### Sequences of primers used (5′ to 3′)

*FLUC*: CAACTGCATAAGGCTATGAAGAGA and ATTTGTATTCAGCCCATATCGTT.

*ACTB*: AAGTGTGACGTTGACATCCG and GATCCACATCTGCTGGAAGG.

*CHAT*: ATGAACGCCTGCCTCCAATCGG and CAGATGCAGCGCTCGATCATG

*RBFOX3:* CTCCAACCCGGCCTCTC and GCACTAGGTTCTCACAGGCA

### Western blotting

Cells were harvested, washed with PBS, and lysed using radioimmunoprecipitation assay lysis buffer (50 mM Tris–HCl (pH 8.0), 150 mM NaCl, 1 mM sodium deoxycholate, 0.1% SDS, 1% Nonidet P-40) containing protease inhibitor cocktail (Promega). The lysates were subjected to centrifugation at 15,000 rpm for 15 min. Protein concentration was determined using Protein Assay Dye Reagent (Bio-Rad). Seventy to hundred micrograms of lysate was boiled with sample buffer (250 mM Tris–HCl, 10% SDS, 30% (v/v) glycerol, 0.05% (w/v) bromophenol blue) at 95 °C for 5 min. The samples were then treated with 100 mM DTT for 15 min and subjected to denaturing electrophoresis in 10% (for SERCA2) or 15% (for other proteins) SDS-polyacrylamide gel. The resolved proteins were transferred onto 0.45 μm polyvinylidene fluoride membrane (for SERCA2) or 0.2 μm polyvinylidene fluoride membrane (for others) using Mini Trans-Blot cell (Bio-Rad). The membrane (Merck) was blocked with 5% Bovine serum albumin (for NNATx) or 3% skimmed milk (for others) for 1 h at room temperature, washed three times, and incubated with the primary antibody overnight at 4 °C. After washing three times with PBS containing 0.1% tween-20, the membrane was incubated with the horseradish peroxidase–conjugated secondary antibody for 2 h at room temperature. The membrane was then washed three times with PBS containing 0.1% tween-20 and developed using Clarity enhanced chemiluminescence reagent (Bio-Rad) or SuperSignal West Femto kit (Thermo Fisher Scientific). Western blotting performed using mouse tissue lysates was approved by our Institutional Animal Ethics Committee. Images were captured using LAS 3000 imager (Fujifilm) or Chemidoc Imaging System (Bio-Rad). Band density was quantified using ImageJ software (https://imagej.nih.gov/ij/), and the density values were normalized to that of loading control (actin).

### Ribosome profiling data analysis

Ribosome profiling datasets were retrieved from National Center for Biotechnology Information database using Sequence Read Archive Toolkit. The FASTQ files were processed, and the adaptor sequences were removed using fastp tool. Reads with a minimum length of 24 nucleotides and 100% match with the reference transcript of *NNAT* (NM_010923.3) were mapped onto different regions (5′ UTR, CDS, and 3′ UTR) of *NNAT* mRNA as described previously (36). Reads aligning to the region starting from 12 nucleotides upstream of the first stop codon and ending at 22 nucleotides upstream of the second stop codon were assigned to the ISR. The reads aligning to the rest of the 3′ UTR were assigned to the 3′ UTR. Ribosome footprint density per nucleotide was calculated as number of reads in each region divided by the length of the region (*i.e.*, ISR or 3′ UTR).

### Overexpression of NNAT and NNATx

The CDSs of NNAT and NNATx were cloned in pcDNA3.1 backbone. In case of NNATx, the canonical stop codon of *NNAT* was changed to UCA to ensure its maximum expression. Two micrograms of these constructs were transfected in Neuro-2a cells seeded in a 6-well plate. After 48 h, they were selected using geneticin (0.5 mg/ml, Sigma) for a week to obtain stably expressing cells, which were then maintained in the medium described above with 0.2 mg/ml of geneticin. Expression was confirmed by Western blot using anti-NNAT or anti-NNATx antibody.

### shRNA-mediated knockdown

Neuro-2a cells were transfected with shRNA constructs (Sigma) using Lipofectamine 2000, and stable cells were obtained after puromycin (2 μg/ml) selection. Knockdown was confirmed by Western blotting or qRT-PCR.

*NNAT* shRNA1: Clone ID: TRCN0000159729.

*NNAT* shRNA2: Clone ID: TRCN0000163961.

*NONO* shRNA1: Clone ID: TRCN0000074558.

Control shRNA: MISSION pLKO.1-puro Non-Mammalian shRNA Control Plasmid.

### Measurement of cytoplasmic Ca^2+^

Cytoplasmic Ca^2+^ was measured using Fluo-4 AM (Invitrogen, F14217), a cell-permeant green-fluorescent high-affinity calcium indicator. Cells were seeded at a density of 25,000 cells/well in a 96-well plate. Eight hours after seeding, medium was changed to differentiation medium. After 24 h, cells were treated with 2 μM Fluo-4 AM in MEM without serum and antibiotics for 1 h. Cells were then washed and incubated for 30 min in Hanks’ Balanced Salt Solution. Fluo-4 fluorescence was measured in a microplate reader (Infinite M200 PRO, TECAN) at an excitation wavelength of 480 nm and emission wavelength of 520 nm, respectively. In one set, 2 mM of EGTA, a selective calcium ion chelator, was used to ensure that the fluorescent signal was due to Ca^2+^.

Live-cell imaging for cytoplasmic Ca^2+^ influx was performed using Olympus FLUOVIEW FV3000 confocal microscope (objective: UPlanSApo 60×/1.35 oil ∞/0.17/FN26.5). Two micromolar retinoic acid was added to Neuro-2a cells seeded in a confocal dish just before the image acquisition. Total 750 images were captured at a frame rate of 30 images per second.

### Immunoprecipitation

Cells were lysed in lysis buffer (50 mM Tris–HCl (pH 7.4), 150 mM NaCl, 2 mM MgCl_2_, 0.1% Nonidet-P40). Lysate containing 700 μg of protein was incubated with anti-FLAG M2 Affinity Gel (A2220, Sigma) overnight with gentle agitation at 4 °C. The beads were centrifuged and washed with Tris-buffered saline. The bound proteins were extracted by heating the beads with Laemmli sample buffer (without DTT) at 95 °C for 10 min. The beads were then treated with 100 mM DTT for 15 min and subjected to Western blotting.

### Neurite length analysis

Cells were seeded (5000 cells/well) in a 12-well plate and differentiation was induced as described above. Phase contrast microscopy images were captured using Olympus IX73 microscope (objective: CPlan N 10x/0.25 PhC ∞/-/FN22). Neurites protruding from the cell bodies were traced semiautomatically using Simple Neurite Tracer plugin of ImageJ software with default settings (60). Cells were counted using Cell Counter plugin of ImageJ. Total length of the neurites traced were normalized with the number of cells showing neurite outgrowth for each image to calculate neurite length/cell.

### Transfection with ASOs

Neuro-2a cells were seeded in a 6-well plate. At 60% to 70% confluency, cells were transfected with 5 μM ASOs with Lipofectamine 2000 (Invitrogen) in Opti-MEM medium (Thermo Fisher Scientific). Eight hours after transfection, media was changed to complete medium (MEM) with 10% FBS. After 12 h, cells were treated with differentiation medium containing 2 μM retinoic acid for 24 h. After this, cells were used for Western blotting, quantitative real-time PCR, TCR assay, and Ca^2+^ measurement.

Sequences of DNA ASOs (5′-3′):

Control oligo (targets the CDS of GFP): GGAAAATCTATCCAAAATTCTGCAG.

+43 ASO: CCCATGCTGGTCGAGAAGCACAGG.

These ASOs were Cy3 modified at the 5′ end for fluorescence imaging and flow cytometry analysis.

### Generation of NNATx-deficient cells using CRISPR-Cas9 system

The sgRNAs (5′-CCCCAGCTCCCAGCCCTGGG-3′ and 5′-CACTTGCCAAGGTCA GTGAG-3′) were cloned in pSpCas9(BB)-2A-GFP (PX458) plasmid. Two micrograms of each sgRNA-expressing plasmid were transfected in Neuro-2a cells at 75% confluence in a 35-mm dish using Lipofectamine 2000. After 48 h, the cells positive for GFP were sorted and seeded at a density of one cell per well in a 96-well plate (FACSAria II sorter from BD Biosciences). Cells were allowed to proliferate, and they were screened for the deletion/mutation in the ISR of *NNAT* by PCR (5′-TTCAGGTACTCCCTGCAGAA-3′ and 5′-CTACGCCCATATCTCGAGGG-3′), followed by sequencing of the PCR product and then by Western blotting.

## Data availability

All data are contained within the manuscript.

## Supporting information

This article contains supporting information.

## Conflict of interest

The authors declare that they have no conflicts of interest with the contents of the article.

## References

[1] Pitale P.M., Howse W., Gorbatyuk M. (2017). Neuronatin protein in health and disease. J. Cell Physiol..

[2] Joseph R., Dou D., Tsang W. (1994). Molecular cloning of a novel mRNA (neuronatin) that is highly expressed in neonatal mammalian brain. Biochem. Biophys. Res. Commun..

[3] Joseph R., Dou D., Tsang W. (1995). Neuronatin mRNA: alternatively spliced forms of a novel brain-specific mammalian developmental gene. Brain Res..

[4] Lin H.H., Bell E., Uwanogho D., Perfect L.W., Noristani H., Bates T.J. (2010). Neuronatin promotes neural lineage in ESCs via Ca(2+) signaling. Stem Cells.

[5] Britzolaki A., Saurine J., Flaherty E., Thelen C., Pitychoutis P.M. (2018). The SERCA2: a gatekeeper of neuronal calcium homeostasis in the brain. Cell Mol. Neurobiol..

[6] Oyang E.L., Davidson B.C., Lee W., Poon M.M. (2011). Functional characterization of the dendritically localized mRNA neuronatin in hippocampal neurons. PLoS One.

[7] Suh Y.H., Kim W.H., Moon C., Hong Y.H., Eun S.Y., Lim J.H. (2005). Ectopic expression of Neuronatin potentiates adipogenesis through enhanced phosphorylation of cAMP-response element-binding protein in 3T3-L1 cells. Biochem. Biophys. Res. Commun..

[8] Vatsa N., Kumar V., Singh B.K., Kumar S.S., Sharma A., Jana N.R. (2019). Down-regulation of miRNA-708 promotes aberrant calcium signaling by targeting neuronatin in a mouse model of Angelman Syndrome. Front. Mol. Neurosci..

[9] Joe M.K., Lee H.J., Suh Y.H., Han K.L., Lim J.H., Song J. (2008). Crucial roles of neuronatin in insulin secretion and high glucose-induced apoptosis in pancreatic beta-cells. Cell Signal..

[10] Millership S.J., Da Silva Xavier G., Choudhury A.I., Bertazzo S., Chabosseau P., Pedroni S.M. (2018). Neuronatin regulates pancreatic beta cell insulin content and secretion. J. Clin. Invest..

[11] Chu K., Tsai M.J. (2005). Neuronatin, a downstream target of BETA2/NeuroD1 in the pancreas, is involved in glucose-mediated insulin secretion. Diabetes.

[12] Mzhavia N., Yu S., Ikeda S., Chu T.T., Goldberg I., Dansky H.M. (2008). Neuronatin: a new inflammation gene expressed on the aortic endothelium of diabetic mice. Diabetes.

[13] Gburcik V., Cleasby M.E., Timmons J.A. (2013). Loss of neuronatin promotes "browning" of primary mouse adipocytes while reducing Glut1-mediated glucose disposal. Am. J. Physiol. Endocrinol. Metab..

[14] Vrang N., Meyre D., Froguel P., Jelsing J., Tang-Christensen M., Vatin V. (2010). The imprinted gene neuronatin is regulated by metabolic status and associated with obesity. Obesity.

[15] Sharma J., Mukherjee D., Rao S.N., Iyengar S., Shankar S.K., Satishchandra P. (2013). Neuronatin-mediated aberrant calcium signaling and endoplasmic reticulum stress underlie neuropathology in Lafora disease. J. Biol. Chem..

[16] Nass N., Walter S., Jechorek D., Weissenborn C., Ignatov A., Haybaeck J. (2017). High neuronatin (NNAT) expression is associated with poor outcome in breast cancer. Virchows Archiv..

[17] Xu D.S., Yang C., Proescholdt M., Brundl E., Brawanski A., Fang X. (2012). Neuronatin in a subset of glioblastoma multiforme tumor progenitor cells is associated with increased cell proliferation and shorter patient survival. PLoS One.

[18] Ryu S., McDonnell K., Choi H., Gao D., Hahn M., Joshi N. (2013). Suppression of miRNA-708 by polycomb group promotes metastases by calcium-induced cell migration. Cancer Cell.

[19] Dinman J.D. (2012). Control of gene expression by translational recoding. Adv. Protein Chem. Struct. Biol..

[20] Hill C.H., Brierley I. (2023). Structural and functional Insights into viral programmed ribosomal frameshifting. Annu. Rev. Virol..

[21] Floquet C., Hatin I., Rousset J.P., Bidou L. (2012). Statistical analysis of readthrough levels for nonsense mutations in mammalian cells reveals a major determinant of response to gentamicin. PLoS Genet..

[22] Manjunath L.E., Singh A., Som S., Eswarappa S.M. (2022). Mammalian proteome expansion by stop codon readthrough. Wiley Inter. Rev. RNA.

[23] Eswarappa S.M., Potdar A.A., Koch W.J., Fan Y., Vasu K., Lindner D. (2014). Programmed translational readthrough generates antiangiogenic VEGF-Ax. Cell.

[24] Manjunath L.E., Singh A., Sahoo S., Mishra A., Padmarajan J., Basavaraju C.G. (2020). Stop codon read-through of mammalian MTCH2 leading to an unstable isoform regulates mitochondrial membrane potential. J. Biol. Chem..

[25] Singh A., Manjunath L.E., Kundu P., Sahoo S., Das A., Suma H.R. (2019). Let-7a-regulated translational readthrough of mammalian AGO1 generates a microRNA pathway inhibitor. EMBO J..

[26] Yamaguchi Y., Hayashi A., Campagnoni C.W., Kimura A., Inuzuka T., Baba H. (2012). L-MPZ, a novel isoform of myelin P0, is produced by stop codon readthrough. J. Biol. Chem..

[27] Ghosh S., Guimaraes J.C., Lanzafame M., Schmidt A., Syed A.P., Dimitriades B. (2020). Prevention of dsRNA-induced interferon signaling by AGO1x is linked to breast cancer cell proliferation. EMBO J..

[28] Jungreis I., Lin M.F., Spokony R., Chan C.S., Negre N., Victorsen A. (2011). Evidence of abundant stop codon readthrough in Drosophila and other metazoa. Genome Res..

[29] Grentzmann G., Ingram J.A., Kelly P.J., Gesteland R.F., Atkins J.F. (1998). A dual-luciferase reporter system for studying recoding signals. RNA.

[30] Harger J.W., Dinman J.D. (2003). An *in vivo* dual-luciferase assay system for studying translational recoding in the yeast Saccharomyces cerevisiae. RNA.

[31] Bernhart S.H., Hofacker I.L., Will S., Gruber A.R., Stadler P.F. (2008). RNAalifold: improved consensus structure prediction for RNA alignments. BMC Bioinform..

[32] Kar D., Sellamuthu K., Kumar S.D., Eswarappa S.M. (2020). Induction of translational readthrough across the thalassemia-causing premature stop codon in beta-Globin-Encoding mRNA. Biochemistry.

[33] Yu C.H., Noteborn M.H., Olsthoorn R.C. (2010). Stimulation of ribosomal frameshifting by antisense LNA. Nucleic Acids Res..

[34] Ahn D.G., Lee W., Choi J.K., Kim S.J., Plant E.P., Almazan F. (2011). Interference of ribosomal frameshifting by antisense peptide nucleic acids suppresses SARS coronavirus replication. Antivir. Res..

[35] Dinman J.D. (2019). Translational recoding signals: expanding the synthetic biology toolbox. J. Biol. Chem..

[36] Sahoo S., Singh D., Singh A., Pandit M., Vasu K., Som S. (2022). Identification and functional characterization of mRNAs that exhibit stop codon readthrough in Arabidopsis thaliana. J. Biol. Chem..

[37] Dunn J.G., Foo C.K., Belletier N.G., Gavis E.R., Weissman J.S. (2013). Ribosome profiling reveals pervasive and regulated stop codon readthrough in Drosophila melanogaster. eLife.

[38] Katz Y., Li F., Lambert N.J., Sokol E.S., Tam W.L., Cheng A.W. (2014). Musashi proteins are post-transcriptional regulators of the epithelial-luminal cell state. eLife.

[39] Fujii K., Shi Z., Zhulyn O., Denans N., Barna M. (2017). Pervasive translational regulation of the cell signalling circuitry underlies mammalian development. Nat. Commun..

[40] Belew A.T., Meskauskas A., Musalgaonkar S., Advani V.M., Sulima S.O., Kasprzak W.K. (2014). Ribosomal frameshifting in the CCR5 mRNA is regulated by miRNAs and the NMD pathway. Nature.

[41] Kwak H., Park M.W., Jeong S. (2011). Annexin A2 binds RNA and reduces the frameshifting efficiency of infectious bronchitis virus. PLoS One.

[42] Charbonneau J., Gendron K., Ferbeyre G., Brakier-Gingras L. (2012). The 5' UTR of HIV-1 full-length mRNA and the Tat viral protein modulate the programmed -1 ribosomal frameshift that generates HIV-1 enzymes. RNA.

[43] Pan X., Fang Y., Li X., Yang Y., Shen H.B. (2020). RBPsuite: RNA-protein binding sites prediction suite based on deep learning. BMC Genomics.

[44] Knott G.J., Bond C.S., Fox A.H. (2016). The DBHS proteins SFPQ, NONO and PSPC1: a multipurpose molecular scaffold. Nucleic Acids Res..

[45] Furukawa M.T., Sakamoto H., Inoue K. (2015). Interaction and colocalization of HERMES/RBPMS with NonO, PSF, and G3BP1 in neuronal cytoplasmic RNP granules in mouse retinal line cells. Genes Cell.

[46] Kanai Y., Dohmae N., Hirokawa N. (2004). Kinesin transports RNA: isolation and characterization of an RNA-transporting granule. Neuron.

[47] Paredes R.M., Etzler J.C., Watts L.T., Zheng W., Lechleiter J.D. (2008). Chemical calcium indicators. Methods.

[48] Gee K.R., Brown K.A., Chen W.N., Bishop-Stewart J., Gray D., Johnson I. (2000). Chemical and physiological characterization of fluo-4 Ca(2+)-indicator dyes. Cell Calcium.

[49] Leclerc C., Neant I., Moreau M. (2012). The calcium: an early signal that initiates the formation of the nervous system during embryogenesis. Front. Mol. Neurosci..

[50] Toth A.B., Shum A.K., Prakriya M. (2016). Regulation of neurogenesis by calcium signaling. Cell Calcium.

[51] Manabe T., Tatsumi K., Inoue M., Matsuyoshi H., Makinodan M., Yokoyama S. (2005). L3/Lhx8 is involved in the determination of cholinergic or GABAergic cell fate. J. Neurochem..

[52] Personett D., Fass U., Panickar K., McKinney M. (2000). Retinoic acid-mediated enhancement of the cholinergic/neuronal nitric oxide synthase phenotype of the medial septal SN56 clone: establishment of a nitric oxide-sensitive proapoptotic state. J. Neurochem..

[53] Duan W., Zhang Y.P., Hou Z., Huang C., Zhu H., Zhang C.Q. (2016). Novel Insights into NeuN: from neuronal marker to splicing regulator. Mol. Neurobiol..

[54] Wagner N. (2019).

[55] Zhang Z., Carmichael G.G. (2001). The fate of dsRNA in the nucleus: a p54(nrb)-containing complex mediates the nuclear retention of promiscuously A-to-I edited RNAs. Cell.

[56] Kameoka S., Duque P., Konarska M.M. (2004). p54(nrb) associates with the 5' splice site within large transcription/splicing complexes. EMBO J..

[57] Loughran G., Jungreis I., Tzani I., Power M., Dmitriev R.I., Ivanov I.P. (2018). Stop codon readthrough generates a C-terminally extended variant of the human vitamin D receptor with reduced calcitriol response. J. Biol. Chem..

[58] Li W., Karwacki-Neisius V., Ma C., Tan L., Shi Y., Wu F. (2020). Nono deficiency compromises TET1 chromatin association and impedes neuronal differentiation of mouse embryonic stem cells. Nucleic Acids Res..

[59] Knopman D.S., Amieva H., Petersen R.C., Chetelat G., Holtzman D.M., Hyman B.T. (2021). Alzheimer disease. Nat. Rev. Dis. Primers.

[60] Arshadi C., Gunther U., Eddison M., Harrington K.I.S., Ferreira T.A. (2021). SNT: a unifying toolbox for quantification of neuronal anatomy. Nat. Methods.

