# Supplementary material for: Termination codon readthrough of NNAT mRNA regulates calcium-mediated neuronal differentiation
Source: J Biol Chem. 2023 Aug 22;299(9):105184. doi: 10.1016/j.jbc.2023.105184 (PMC10506107; doi:10.1016/j.jbc.2023.105184)
Supplement: Supplemental figures [file mmc1.pdf]

Figure S1

Canonical
stop codon
↑

```
Hs : UGAGGCCCCAGCUCCCAGCCCUGGGCGGCCGUAUCAUCAGGUGCUCCUGUGCAUCUCGGCCAGCACGGGAGCCAGUG
Mm : UGAGGCCCCAGCUCCCAGCCCUGGGCGGCCGUAUCAUCAGGUGCUCCUGUGCAUCUCAGCCAGCACGGGAGCCAGUG
Ec : UGAGGCCCCAGCCCCCAGCCCUGGGCGGCCGUGUCACCAGGUGCUCCUGUGCAUCUCGACCAGCAUGGGAGCCAGUG
Bt : UGAGGCCCCAGCCCCUCCCCUGGGCGGCCGUGUCACCAGGUGCUCCUGUGCCAUUCCACCAGCAUGGGAGCCAGCG
Ss : UGAGGCUCCAGCCCCAACCCUGGGCGGCCGUGUCACCAGGUGCUCCUGUGCAAUUCCACCAGCAUGGGAGCCAGUG
Md : UGAGGCCCCAGCCCCCAGCCCUGGGCGGCCAUGUCACCAGGUGCUCCUGUGCAUCUCGCCCAGCAUGGGAGCCAUUG
Fc : UGAGGCCCCAACCCCCAGCCCUGGGCGGCCGCGUCACCAGGUGCUCCUGUGCUUCUCGGCCAGCAUGGGAGCCAGUG
Mp : UGAGGUCCCAGCCCCCAGCCCUGGGCGGCCUUGUCACCAGGUGCUCCUGUGCUUCUUGGCCAGCAUGGGAGCCAGUG
Ms : UGAGGCCCCAGCUCCCAGCCCUGGGCGGCCGUGUCAUCAGGUGCUCCUGUGCUUCUCGACCAGCAUGGGAGCCAGUG

Hs : CCGCGCAGGAAUGUGGGGUCCCCUGUGUUCCCUCGCCAGAGGAGCACUUGGCAAGGUCAGUGAGGGGCCAGUAGACC
Mm : CCGCACAGGAAUGGGGGGUCCCCUGUGCUCCCUCGCCAGAGGAGCACUUGGCAAGGUCAGUGAGGGGCCAGUAGACC
Ec : CCGCGCAGGAACGGGGGGUCCCCUGUGCUCCCUCGCCAUAGGAGCACUUGCCAAGGUCAGUGAGGGGCCGGUAGGCC
Bt : CCGCGCAGGAAUGGGGCGUCCCCUGUGCCCUCUCGCCAGAGGAGCACUUGCCAAGGUCAGUGAGGGGCUGGUAGGCC
Ss : CCGCACAGGAACGGGGCGUCCCCUGUGCCCUCUCGCCAGAGGAGCACUUGCCAAGGUCAGUGAGGGGCUGGUAGGCC
Md : CCGCCCAGGAAUAGGGGGUCCCCUGUGCUCUCUCGCCAGAGGAGCAUUUGCCAAGGUCAAUGAGGGGCUGGCAGGCC
Fc : CCGCGCAGGAAUGGGGGGUCCCCUGUGCUCUCUCGCCAGAGGAGCAUUUGCCAAGGUCAGUGAGGGGCCGGUAGGCC
Mp : CCGCGCAGGAAUGGGGGGUCCCCUGUGCUCUCUCGCCAGAGGAGCAUUUGCCAAGGUCAGUGAGGGGCUGGUAGGCU
Ms : CCGCGCAGGAAUGGGGGGUCCCCUGUGUUCCCUCGUCAGAGGAGCACUUGCCAAGGUCAGUGAGGGGCCGGUAGGCC

Hs : CCCGGAGAAGCAGUACCGACAAUGACGAAGAUACCAGAUCCCUUCCCAACCCCUUUGCACCGGUCCCACUAAGGGGC
Mm : CCUGGAGAAGCAGUACCGACAAUGACGAAGAUACCAGAUCCCUUCCCAACCCCUUUGCACCAGUCCCACCAAGGGGC
Ec : CCCGGAGAAGCAGCACCGACAAUGAUGACAAUACCAGAACCUUUCCAAACCCCUCUGCACCCGUCCCACUGCCAGGC
Bt : CCUGGAGAAGCAGCACCGACAAUGACGACAACAAGAGAUCCCUUCCCCACCCCUCUGCACCCCUCCCACUGCGGGUG
Ss : CCCAGAGAAGCAGCACCGACAAUGACGACAAUACCAGAUUCCUUCCCCACCCCUUUGCACCCAUCCUGCUGCCGGUG
Md : UCCAGAGAAGCAGCACCGCAAUGACGACAAUACCUGAUCCCUUUCCAAUCCCUGUGUACCCCUCCCAUUAUGGGGCG
Fc : CCCGGAGAAGCAGCACCGACAAUGACGACAAUACCAGAUCCCUUCCAAACCCCUUUGCGCCCCUCUGACCACUGGGU
Mp : CCUGGAGAAGCAGCACCGACAAUGACGACAAUACCAGAUCCCUUCCAAGCCCCUUUGCACCCCUCUGACUGCUGGGU
Ms : CCCAGAAAAGCAGCACCGACAAUGAUGAAGAUAUCAGUUCCCUUCCCAGCCCCUUUGCCCCUGUCCCACUACCGGCG

Hs : AGGGUCGAGAGAGGAGGGGGGAUAGGGGGAGCAGACCCCUGAGAUCUGGGC
Mm : AGGGUCGGGAGAGGAGGGGGGAUAGGGGGAGCAGACCCCUGAGAUUUGGGC
Ec : GGGGUAGAGGGAGGAGGGGGGUUUGGGGGAGCAGACCCCUGAGAUCUGGGC
Bt : GCCUAGAGGGAGGAACGGGGCUUAGGGGGAGCAGACUCCUGAGAUCUGGGC
Ss : GCAUAGAGGGAGGAGGGGGGAAUGGAGGGAGCAGACCCCUGAGAUCUGGGC
Md : GGGUAGAGGGAGGAGGGAUGGAGGGAGAAUUCUGAGAUUUGGGCAUAGGCA
Fc : GGGGUAGAGGGAGGAGGGGGGUGGGGGGAGCAGACCCCCGAGAUCUGGGCA
Mp : GGGGUAGAGGGAGGAGGGGGGGUGGGGGGGAGCUUACCCCCAAGAUCUGGG
Ms : GGUGGGAGAGGAGGGGGGAAGAGGGGAGCAACCCUCGAGAUAUGGGCGUAG
```

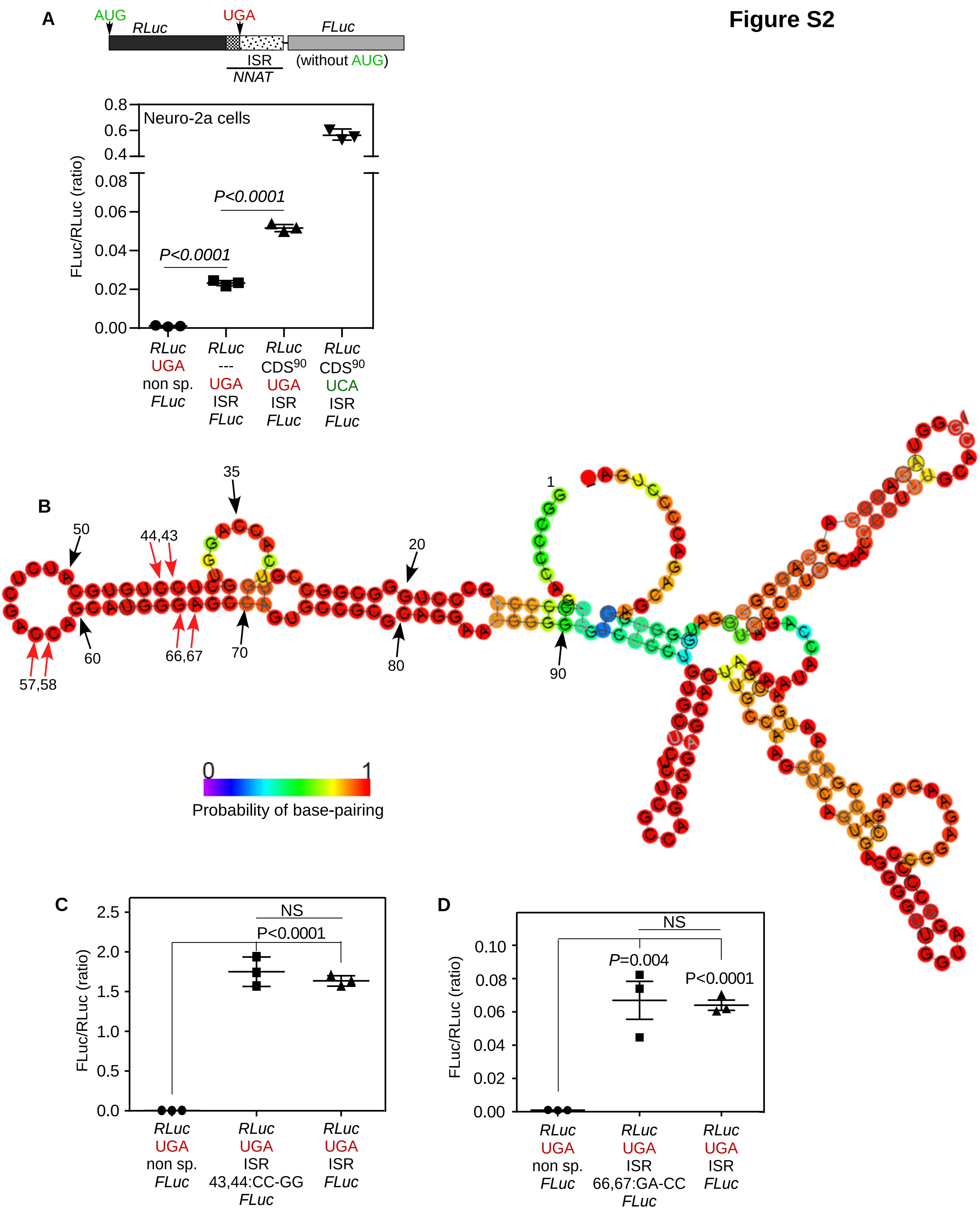

Figure S2
A
AUG
RLuc
UGA
FLuc
ISR
NNAT
(without AUG)
Neuro-2a cells
FLuc/RLuc (ratio)
0.8
0.6
0.4
0.08
0.06
0.04
0.02
0.00
P<0.0001
P<0.0001
RLuc UGA non sp. FLuc
RLuc --- UGA ISR FLuc
RLuc CDS90 UGA ISR FLuc
RLuc CDS90 UCA ISR FLuc
B
1
20
35
44,43
50
57,58
60
66,67
70
80
90
0
1
Probability of base-pairing
C
FLuc/RLuc (ratio)
2.5
2.0
1.5
1.0
0.5
0.0
NS
P<0.0001
RLuc UGA non sp. FLuc
RLuc UGA ISR 43,44:CC-GG FLuc
RLuc UGA ISR FLuc
D
FLuc/RLuc (ratio)
0.10
0.08
0.06
0.04
0.02
0.00
NS
P=0.004
P<0.0001
RLuc UGA non sp. FLuc
RLuc UGA ISR 66,67:GA-CC FLuc
RLuc UGA ISR FLuc

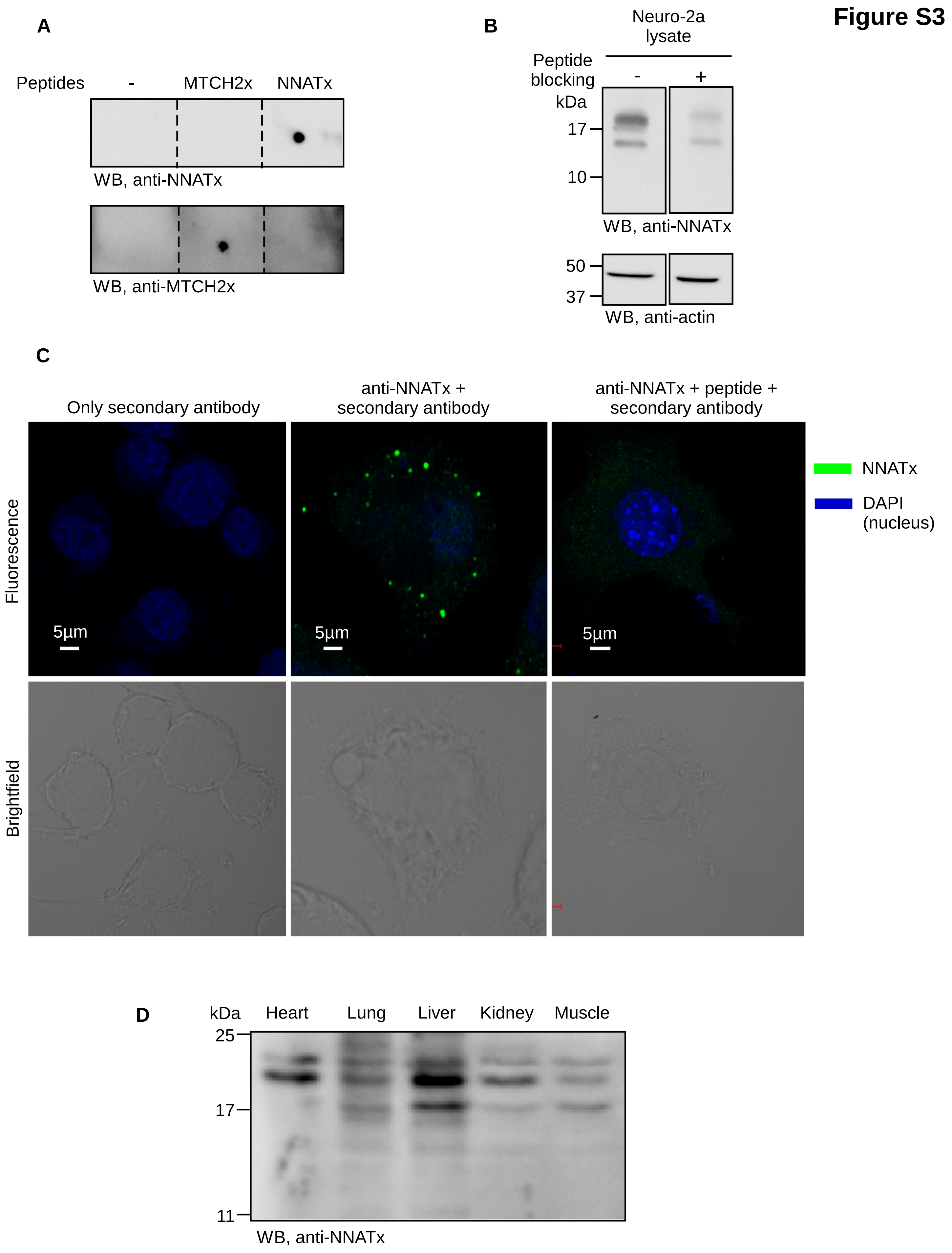
Figure S3
A
Peptides
-
MTCH2x
NNATx
WB, anti-NNATx
WB, anti-MTCH2x
B
Neuro-2a
lysate
Peptide
blocking
-
+
kDa
17
10
WB, anti-NNATx
50
37
WB, anti-actin
C
Only secondary antibody
anti-NNATx +
secondary antibody
anti-NNATx + peptide +
secondary antibody
Fluorescence
Brightfield
5µm
5µm
5µm
NNATx
DAPI
(nucleus)
D
kDa
Heart
Lung
Liver
Kidney
Muscle
25
17
11
WB, anti-NNATx

Figure S4

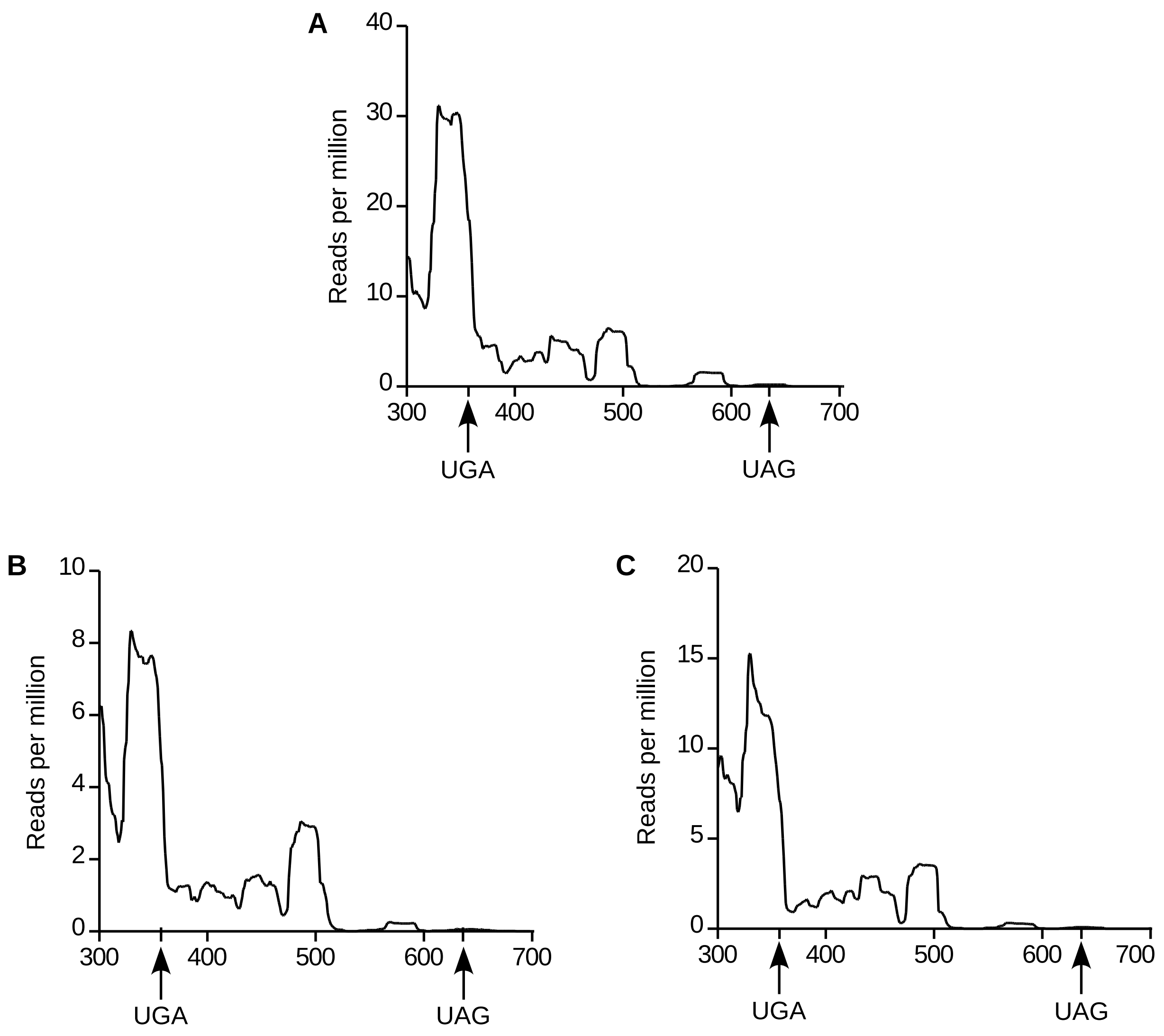
A
40
30
20
10
0
Reads per million
300
400
500
600
700
UGA
UAG
B
10
8
6
4
2
0
Reads per million
300
400
500
600
700
UGA
UAG
C
20
15
10
5
0
Reads per million
300
400
500
600
700
UGA
UAG

Figure S5

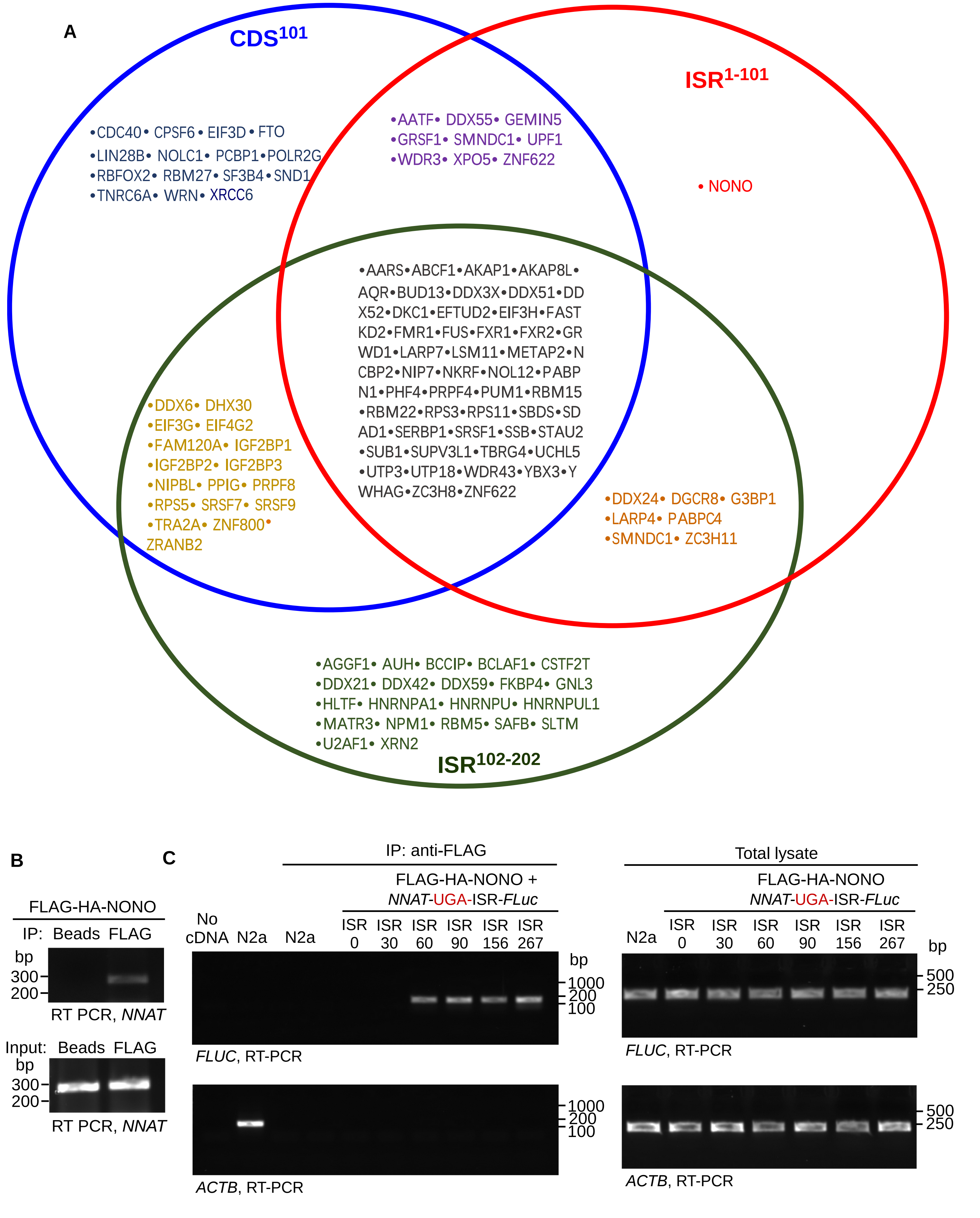
A
CDS101
ISR1-101
ISR102-202
•CDC40• CPSF6• EIF3D• FTO
•LIN28B• NOLC1• PCBP1•POLR2G
•RBFOX2• RBM27• SF3B4• SND1
•TNRC6A• WRN• XRCC6
•AATF• DDX55• GEMIN5
•GRSF1• SMNDC1• UPF1
•WDR3• XPO5• ZNF622
• NONO
•AARS•ABCF1•AKAP1•AKAP8L•
AQR•BUD13•DDX3X•DDX51•DD
X52•DKC1•EFTUD2•EIF3H•FAST
KD2•FMR1•FUS•FXR1•FXR2•GR
WD1•LARP7•LSM11•METAP2•N
CBP2•NIP7•NKRF•NOL12•PABP
N1•PHF4•PRPF4•PUM1•RBM15
•RBM22•RPS3•RPS11•SBDS•SD
AD1•SERBP1•SRSF1•SSB•STAU2
•SUB1•SUPV3L1•TBRG4•UCHL5
•UTP3•UTP18•WDR43•YBX3•Y
WHAG•ZC3H8•ZNF622
•DDX6• DHX30
•EIF3G• EIF4G2
•FAM120A• IGF2BP1
•IGF2BP2• IGF2BP3
•NIPBL• PPIG• PRPF8
•RPS5• SRSF7• SRSF9
•TRA2A• ZNF800•
ZRANB2
•DDX24• DGCR8• G3BP1
•LARP4• PABPC4
•SMNDC1• ZC3H11
•AGGF1• AUH• BCCIP• BCLAF1• CSTF2T
•DDX21• DDX42• DDX59• FKBP4• GNL3
•HLTF• HNRNPA1• HNRNPU• HNRNPUL1
•MATR3• NPM1• RBM5• SAFB• SLTM
•U2AF1• XRN2
B
FLAG-HA-NONO
IP: Beads FLAG
bp
300
200
RT PCR, NNAT
Input: Beads FLAG
bp
300
200
RT PCR, NNAT
C
IP: anti-FLAG
FLAG-HA-NONO +
NNAT-UGA-ISR-FLuc
No cDNA
N2a
N2a
ISR 0
ISR 30
ISR 60
ISR 90
ISR 156
ISR 267
bp
1000
200
100
FLUC, RT-PCR
1000
200
100
ACTB, RT-PCR
Total lysate
FLAG-HA-NONO
NNAT-UGA-ISR-FLuc
N2a
ISR 0
ISR 30
ISR 60
ISR 90
ISR 156
ISR 267
bp
500
250
FLUC, RT-PCR
500
250
ACTB, RT-PCR

Figure S6

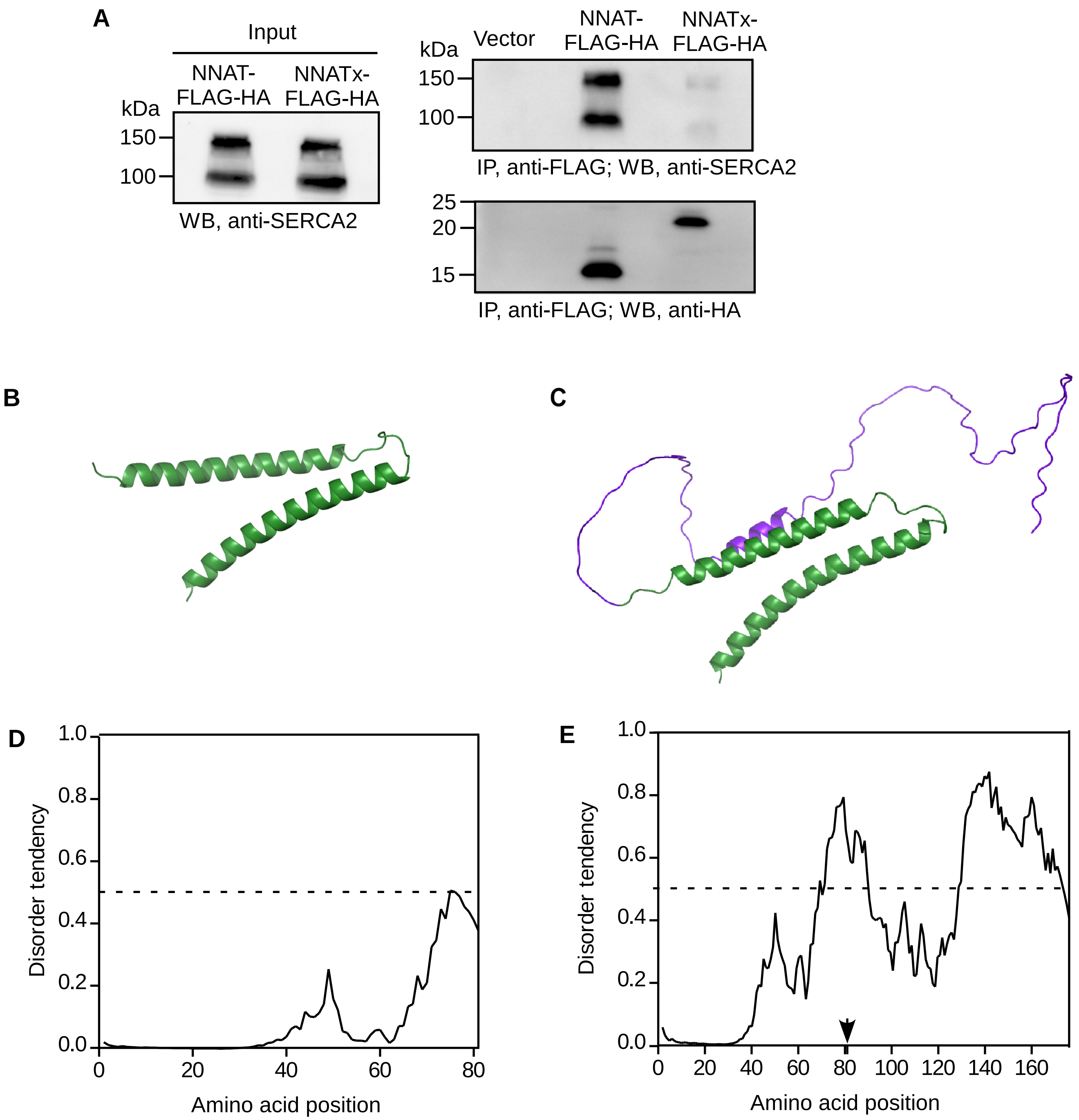

A
Input
NNAT-FLAG-HA
NNATx-FLAG-HA
kDa
150
100
WB, anti-SERCA2
Vector
NNAT-FLAG-HA
NNATx-FLAG-HA
kDa
150
100
IP, anti-FLAG; WB, anti-SERCA2
25
20
15
IP, anti-FLAG; WB, anti-HA
B
C
D
1.0
0.8
0.6
0.4
0.2
0.0
Disorder tendency
0
20
40
60
80
Amino acid position
E
1.0
0.8
0.6
0.4
0.2
0.0
Disorder tendency
0
20
40
60
80
100
120
140
160
Amino acid position

Figure S7

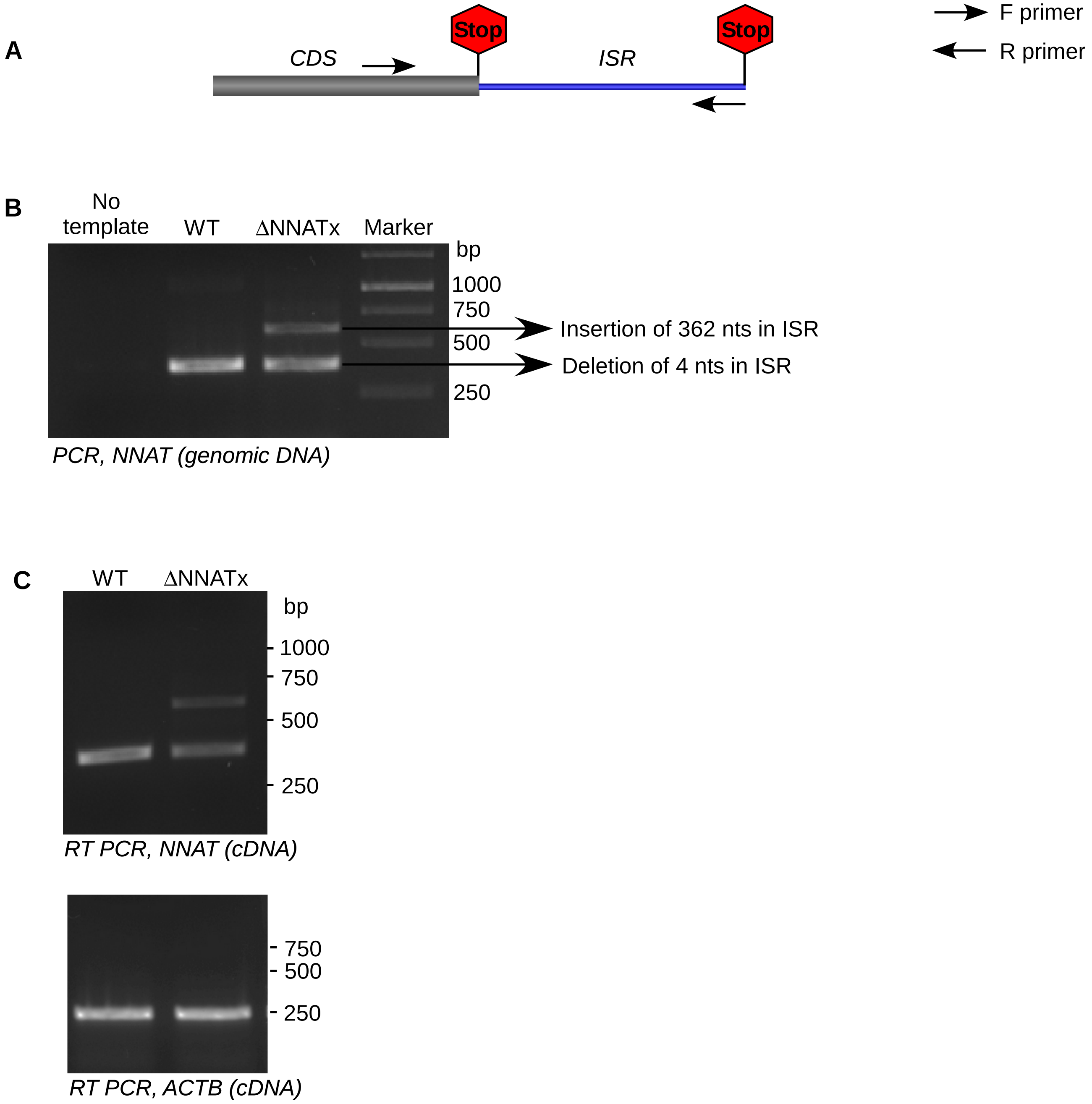

A
Stop
Stop
CDS
ISR
F primer
R primer
B
No template
WT
ΔNNATx
Marker
bp
1000
750
500
250
Insertion of 362 nts in ISR
Deletion of 4 nts in ISR
PCR, NNAT (genomic DNA)
C
WT
ΔNNATx
bp
1000
750
500
250
RT PCR, NNAT (cDNA)
750
500
250
RT PCR, ACTB (cDNA)

Figure S8

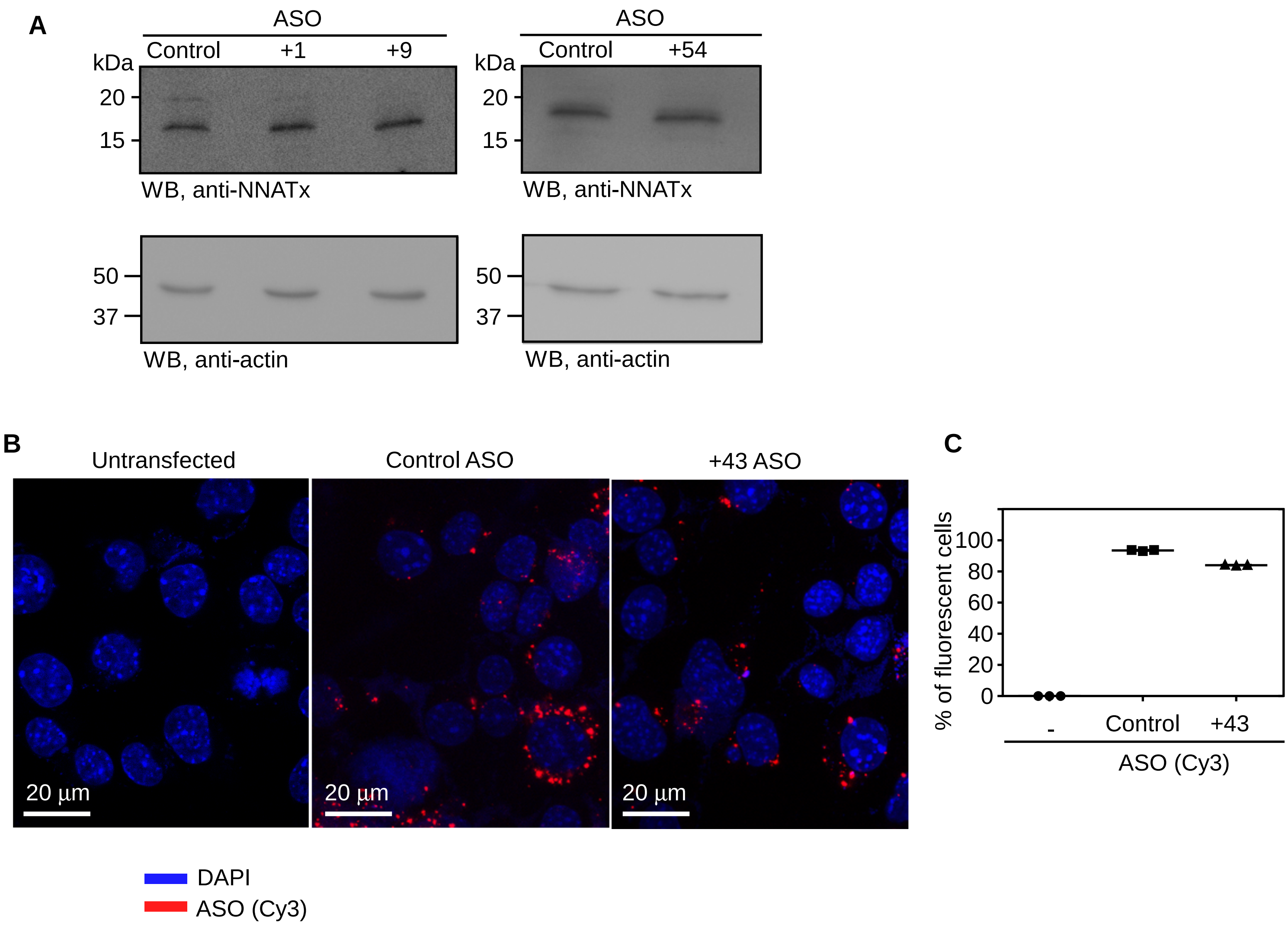
A
ASO
Control
+1
+9
kDa
20
15
WB, anti-NNATx
ASO
Control
+54
kDa
20
15
WB, anti-NNATx
50
37
WB, anti-actin
50
37
WB, anti-actin
B
Untransfected
Control ASO
+43 ASO
20 μm
20 μm
20 μm
DAPI
ASO (Cy3)
C
% of fluorescent cells
100
80
60
40
20
0
-
Control
+43
ASO (Cy3)
