# Supplementary material for: Termination codon readthrough of NNAT mRNA regulates calcium-mediated neuronal differentiation
Source: J Biol Chem. 2023 Aug 22;299(9):105184. doi: 10.1016/j.jbc.2023.105184 (PMC10506107; doi:10.1016/j.jbc.2023.105184)
Supplement: Supporting information [file mmc2.docx]

**Supporting Information**

**Supplementary experimental procedures**

**Sequence alignment**

Nucleotide sequences of 3′UTR of *NNAT* belonging to multiple mammalian species were obtained from NCBI. The sequences were aligned using Clustal Omega. They were *in silico* translated using EMBOSS Transeq tool and the resultant amino acid sequences were aligned using Clustal Omega.

**Construction of plasmids**

The coding sequence (CDS) along with the ISR of *NNAT* was cloned in pcDNA3.1 backbone upstream of and in-frame with the CDS of firefly luciferase (FLuc), between HindIII and BamHI enzyme sites. The second stop codon of *NNAT* and the start codon of *FLuc* were excluded from the construct. A glycine-rich linker sequence (5′-GGCGGCTCCGGCGGCTCCCTCGTGCTCGGG-3′) was cloned between *NNAT* and *FLuc* to minimize any possible interaction between the respective proteins when expressed for readthrough assay. PCR-based site-directed mutagenesis was used to generate mutations.

To generate fluorescence reporter constructs for TCR assay, the CDS of *FLuc* from the above constructs was replaced with that of GFP. Dual luciferase reporter constructs were generated by cloning the ISR and a part of the CDS of *NNAT* (ninety nucleotides upstream of the canonical stop codon) in between the CDSs of *RLuc* and *FLuc* in pcDNA3.1 such that all were in same translation frame. The stop codon of *RLuc* and the start codon of *FLuc* were excluded from the constructs.

**Dot blot analysis**

0.2 μm PVDF (Merck) membrane was activated using methanol and washed according to manufacturer’s protocol. 800 ng of the synthetic peptides (Abgenex) specific to NNATx (SSGAPVHLGQHGSQCR) or MTCH2x (RCGAGTVTFL) were spotted on the membrane. The membrane was maintained undisturbed in a moist chamber at room temperature for 1.5 h and then subjected to Western blot analysis using anti-NNATx or anti-MTCH2x antibody.

**Protein structure prediction**

The structure of NNAT was retrieved from AlphaFold Protein Structure Database. The NNATx structure was predicted based on its amino acid sequence using AlphaFold algorithm (https://colab.research.google.com/github/sokrypton/ColabFold/ blob/main/AlphaFold2.ipynb). Visualization of the structure was done using PyMOL.

**Legends to supplementary figures**

**Figure S1. The proximal 3′UTR of *NNAT* is evolutionarily conserved in mammals.**

Alignment of the nucleotide sequence of the proximal 3′UTR of *NNAT* mRNA belonging to multiple mammalian species. Hs, *Homo sapiens*; Mm, *Macaca mulatta*; Ec, *Equus caballus*; Bt, *Bos taurus*; Ss, *Sus scrofa*; Md, *Myotis davidii*; Fc, *Felis catus*; Mp, *Mustela putorius furo*. Ms, *Mus musculus*. The canonical stop codon and the downstream in-frame stop codons are in red.

**Figure S2. The importance of the coding sequence and the ISR in TCR of *NNAT*.**

(A) Results of dual luciferase-based TCR assay performed in Neuro-2a cells showing the importance of coding sequence (CDS) in TCR of *NNAT*. 90 nucleotides of CDS immediately upstream of the canonical stop codon were included in the TCR reporter (schematic). (B) Putative RNA secondary structure of the ISR as predicted by the RNAalifold webserver**.** Arrows indicate the nucleotide positions. Red arrows indicate the nucleotide positions which were mutated to test TCR activity. (C and D) Results of dual luciferase-based SCR assay in Neuro-2a cells using constructs with mutated ISR (CC to GG in 43^rd^ and 44^th^ positions in (C); GA to CC in 66^th^ and 67^th^ positions in (D)). All graphs (mean ± SD) are representatives of two (A) or three (C and D) independent experiments performed with triplicate samples. Statistical significance was calculated using two-tailed Student’s t-test. Y-axis in all of them represents ratio of FLuc to RLuc activities.

**Figure S3. Specificity of the anti-NNATx antibody.**

(A) Dot blot showing the specificity of anti-NNATx antibody. The peptide, SSGAPVHLGQHGSQCR, specific to NNATx was spotted on the PVDF membrane and anti-NNATx antibody was used to detect the peptide using chemiluminescence. MTCH2x peptide (RCGAGTVTFL) and anti-MTCH2x antibody were used as negative controls for the assay. (B) Western blot showing the expression of NNATx in Neuro-2a cells. To confirm the specificity of the band, the anti-NNATx antibody was pre-incubated with 1 μg/ml of the peptide SSGAPVHLGQHGSQCR for 2 h before using for the Western blot. Two blots (blocked with the peptide and unblocked) were developed at the same time under identical conditions. (C) Immunofluorescence images showing the expression of NNATx in Neuro-2a cells. The anti-NNATx antibody was blocked as mentioned above to confirm the specificity of the signal. Nuclei were visualized by DAPI staining. Green, NNATx (Alexa Fluor 498); Blue, Nucleus (DAPI). (D) Western blot showing the expression of NNATx in multiple organs of a mouse pup.

**Figure S4. Ribosome profiling data provides evidence for TCR of *NNAT*.**

*NNAT* ribosome profile in (A) neural tubes derived from mouse E11.5 embryos (SRR4175367), (B) microdissected forelimb buds from mouse E11.5 embryos (SRR4175373), and (C) cells of mesodermal lineage derived from the mouse primitive streak (SRR4175361). Profiles indicate the presence of ribosomes in the ISR. Positions of the canonical stop codon (UGA) and downstream in-frame stop codon (UAG) are shown.

**Figure S5. NONO protein interacts with *NNAT* mRNA.**

(A) Venn diagram showing the proteins that are predicted to bind ISR^1-101^ (first 101 nucleotides of *NNAT* ISR), CDS^101^ (3′ 101 nucleotides of *NNAT* CDS) and ISR^102-202^ (101 nucleotides from the second half of the *NNAT* ISR). The prediction was performed using RBPsuite, a deep learning-based webserver. (B) Semi-quantitative RT-PCR result showing the enrichment of *NNAT* mRNA along with immunoprecipitated FLAG-HA-NONO (see Fig 4B). Immunoprecipitation from transfected Neuro-2a cells was performed using anti-FLAG M2 affinity gel. (C) RT-PCR results performed to identify the location in the ISR of *NNAT* that interacts (directly or indirectly) with NONO. Neuro-2a (N2a) cells were transfected with FLAG-HA-tagged NONO construct and *NNAT* constructs with multiple deletions in the ISR. ISR numbers indicate the length of the ISR stretch (from 5′ end) used in the assay. NONO was immunoprecipitated using anti-FLAG beads and the precipitate was used for RNA extraction followed by RT-PCR of *NNAT* mRNA. N2a, Neuro-2a cells.

**Figure S6. NNAT isoforms and their interaction with SERCA2**

(A) Unlike NNAT, NNATx does not interact with SERCA2 in HEK293 cells. Co-immunoprecipitation assay was performed in HEK293 cells to test the interaction of NNAT-FLAG-HA and NNATx-FLAG-HA with SERCA2. (B) The structure of mouse NNAT retrieved from the AlphaFold Protein Structure Database. (C) The structure of mouse NNATx predicted using AlphaFold algorithm. The amino acid sequence encoded by the canonical CDS is shown in green and that by the ISR is shown in purple. (D and E) Disorder tendency of mouse NNAT (D) and NNATx (E) estimated using IUPred2A, a web-based tool that predicts intrinsically disordered regions (https://iupred2a.elte.hu/). The position of the canonical stop codon (82) is indicated by an arrow.

**Figure S7. *NNAT* in TCR-deficient (ΔNNATx) cells**

(A) Schematic showing the approximate location of the primers used in genomic DNA PCR reactions performed to screen for ΔNNATx cells. (B) Results of genomic DNA PCR performed using those primers (5′-TTCAGGTACTCCCTGCAGAA-3′ and 5′-CTACGCCCATATCTCGAGGG-3′). Insertion and deletion observed after sequencing are indicated. (C) Results of RT-PCR using the same set of primers showing the expression of *NNAT*.

**Figure S8. Effect of ASOs on the expression of NNATx**

(A) Western blot showing the expression of NNATx in Neuro-2a cells transfected with indicated ASOs. Assay was performed 24 h after the transfection. Sequences of ASOs (5′ - 3′): +1 - CCGCCCAGGGCTGGGAGCTGGGGCC; +9 -ATGATACGGCCGCCCAGGGCTGGGA; +54 - GCACTGGCTCCCATGCTGGTCG; control - GGAAAATCTATCCAAAATTCTGCAG. (B) Images of Neuro-2a cells transfected with Cy3 labelled control and +43 ASOs. (C) Same cells were subjected to flow cytometry for quantification of Cy3+ve cells.
